# *Francisella tularensis* universal stress protein contributes to persistence during growth arrest and paraquat-induced superoxide stress

**DOI:** 10.1128/jb.00377-24

**Published:** 2025-01-23

**Authors:** Benjamin Girardo, Yinshi Yue, Oksana Lockridge, Amanda M. Bartling, Lawrence M. Schopfer, Leonardo Augusto, Marilynn A. Larson

**Affiliations:** 1 Department of Pathology, Microbiology, and Immunology, University of Nebraska Medical Center 12284 , Omaha, Nebraska, USA; 2 Eppley Institute, University of Nebraska Medical Center 12284 , Omaha, Nebraska, USA; NCBI, NLM, National Institutes of Health, Bethesda, Maryland, USA

**Keywords:** *Francisella tularensis*, tularemia, universal stress protein (Usp), oxidative stress defense, reactive oxygen species, superoxide anions, paraquat, catalase, growth arrest

## Abstract

*Francisella tularensis* is one of the most virulent bacterial pathogens known and causes the disease tularemia, which can be fatal if untreated. This zoonotic and intracellular pathogen is exposed to diverse environmental and host stress factors that require an appropriate response to survive. However, the stress tolerance mechanisms used by *F. tularensis* to persist are not fully understood. To address this aspect, we evaluated the highly conserved universal stress protein (Usp) that is encoded by a single-copy gene in *F. tularensis*, unlike the majority of other bacterial pathogens that produce several to many Usp homologs. We determined that the *F. tularensis* Usp transcript is unusually stable with a half-life of over 30 minutes, and that *usp* transcript and protein levels remained abundant when exposed to low pH, nutrient deprivation, hydrogen peroxide, and paraquat. Of these and other stress conditions evaluated, the *F. tularensis* Δ*usp* mutant only exhibited reduced survival relative to the wild type during stationary phase and exposure to paraquat, a highly toxic compound that generates superoxide anions and other free radicals. Comparison of transcript levels in untreated and paraquat-treated *F. tularensis* wild type and Δ*usp* indicated that Usp contributes to enhanced expression of antioxidant defense genes, *oxyR* and *katG*. In summary, the high abundance and stability of Usp provide prompt protection during extended periods of growth arrest and free radical exposure, promoting *F. tularensis* persistence. We propose that *F. tularensis* Usp contributes to an adaptive response that prolongs viability and increases the longevity of this zoonotic pathogen in the environment.

**IMPORTANCE:**

*Francisella tularensis* is classified as a Tier 1 select agent due to the low infectious dose, ease of transmission, and potential use as a bioweapon. To better understand the stress defense mechanisms that contribute to the ability of this highly virulent pathogen to persist, we evaluated the conserved *F. tularensis* universal stress protein (Usp). We show that *F. tularensis* Usp is unusually stable and remains abundant, regardless of the stress conditions tested, differing from other bacterial Usp homologs. We also determined that *F. tularensis* Usp enhances the expression of several critical antioxidant defense genes and increases survival during paraquat exposure and growth arrest. Determining the factors that promote *F. tularensis* persistence in the environment is needed to prevent tularemia transmission.

## INTRODUCTION

*Francisella tularensis* is a highly infectious pathogen, which causes the zoonotic disease tularemia (1). This gram-negative bacterium has been isolated from at least 300 different species across various taxa, including mammals (e.g., humans, rabbits, cats, mice, etc.), invertebrates (e.g., ticks, mosquitoes, deer flies, etc.), birds, amphibians, fish, reptiles, and amoeba (2, 3). Although *F. tularensis* infects both phagocytic and non-phagocytic cells, macrophages are the primary site of replication (4–6). Tularemia in humans occurs via a bite by an infected animal or arthropod, ingestion of contaminated water or food, or inhalation of aerosolized *F. tularensis* (1). Due to the low inoculum dose, ease of dissemination, and prior use as a biological weapon, *F. tularensis* has been classified as a Tier 1 select agent by the Centers for Disease Control and Prevention (CDC). *F. tularensis* subspecies *tularensis* (also referred to as type A) and subspecies *holarctica* (also referred to as type B) are the two subpopulations that primarily cause tularemia and are predominantly located in North America or throughout the Northern hemisphere, respectively (7). Due to additional genomic alterations and subsequent phenotypic differences, the *F. tularensis* type A clade is further subdivided into subtypes A.I and A.II, with the A.I strains being some of the most pathogenic bacteria known (8, 9).

Highly conserved genes encoding universal stress protein (Usp) domains are found throughout the three domains of life, specifically Archaea, Bacteria, and Eukarya, with the exception of humans (10–12). Proteins containing these ubiquitous domains are typically upregulated during stress, suggesting an important biological role (13). Usp was first discovered in *Escherichia coli* by Nyström and Neidhardt and consequently identified as UspA. This single Usp domain protein was found to be produced during growth arrest due to the lack of nutrients or due to the presence of acids, oxidants, antibiotics, and heavy metals (14). In addition to UspA, *E. coli* produces four other single domain Usp paralogs, specifically UspC, UspD, UspF, and UspG, as well as the two Usp domain paralogs, UspE (15). These six *E. coli* proteins are all considered members of the universal stress protein family A superfamily with the identifier IPR006015 in the InterPro database (16). The National Center for Biotechnology Information (NCBI) Conserved Domain Tool utilizes another classification for Usp families, some of which are Usp-like with identifier cd00293 or Usp/KdpD-like with identifier cd01987 (17). The Pfam database classifies these proteins as part of the universal stress protein family with the identifier PF00582 and contains over 205,000 entries (18). Therefore, inconsistencies exist in Usp nomenclature and categorization, which are based on amino acid sequence, domain organization, and/or the ability to bind nucleotides, such as ATP or cAMP (11, 19, 20).

Depending upon the Usp homolog and organism, this family of proteins has been implicated in various functions that promote survival during external stress (10, 11, 21). In bacteria, these roles include oxidative and heat shock defense, survival during nutrient starvation, cell adhesion and motility, pathogenesis, acetylation activities, biofilm formation, and protein complex stabilization (10, 14, 15, 22–25). In *Mycobacterium tuberculosis*, an ATP-binding Usp paralog (locus tag Rv2623) appears to have a species-specific role that is required for the transition from latent to chronic tuberculosis (26). The majority of bacterial Usp research has focused on the six *E. coli* and nine *M. tuberculosis* Usp homologs, complicating data interpretation due to potential overlapping function(s) (10). Although most bacterial pathogens encode several Usp homologs, *F. tularensis* encodes a single *usp* gene that is highly conserved in all the subpopulations. We hypothesized that Usp provides protection to *F. tularensis* during unfavorable conditions, allowing the zoonotic and intracellular pathogen *F. tularensis* to persist in diverse and ever-changing environments. We previously determined that *F. tularensis* Usp is post-translationally modified when recombinantly expressed in *E. coli* (27). In the current study, we demonstrate that the *F. tularensis* Usp transcript is monocistronic and stable with a half-life of over 30 min. We show that *F. tularensis* Usp mRNA and protein levels remain abundant, regardless of the absence or presence of stress factors, in contrast to the regulation of other bacterial Usp homologs that have been studied to date. *In vitro* comparisons of a markerless and in-frame *usp* deletion mutant (Δ*usp*) relative to wild type (WT) revealed that *F. tularensis* Usp contributes to survival during stationary phase and superoxide stress, as well as importantly promotes the expression of oxidative defense genes, *oxyR* and *katG*. These findings demonstrate that *F. tularensis* Usp enhances the ability of this zoonotic pathogen to withstand nutrient limitation and free radical exposure, which likely promotes persistence in the environment.

## RESULTS

### *F. tularensis* Usp genetic organization

Initial chromosomal evaluations of *F. tularensis* Usp included a comparison of the associated gene and adjacent genes in three clinically relevant clades, specifically subtypes A.I and A.II and type B. As shown in Fig. S1, the gene encoding *usp* in all *F. tularensis* subpopulations is 837 bp in length and adjacent to a gene encoding a DEAD/DEAH box helicase, which participates in RNA metabolism (28). A pseudogene, followed by a gene encoding the insertion sequence (IS) element IS*Ftu1*, flanks *usp* in all the clades (Fig. S1). Of note, IS*Ftu1* is the most abundant IS element in *F. tularensis* genomes with over 45 chromosomal copies and appears to be the primary driving force contributing to genomic decay and clade-specific rearrangements (29–31). Since the A.I, A.II, and B clades each differ in genetic content after this IS*Ftu1*, a translocation event apparently occurred in this region that contributed to subspeciation (Fig. S1). However, the retention of *usp* and the adjacent gene encoding a DEAD/DEAH box helicase in all the *F. tularensis* subpopulations suggests that these gene products contribute to the fitness of this pathogen.

### *F. tularensis* Usp domains and amino acid content

**Fig 1:**
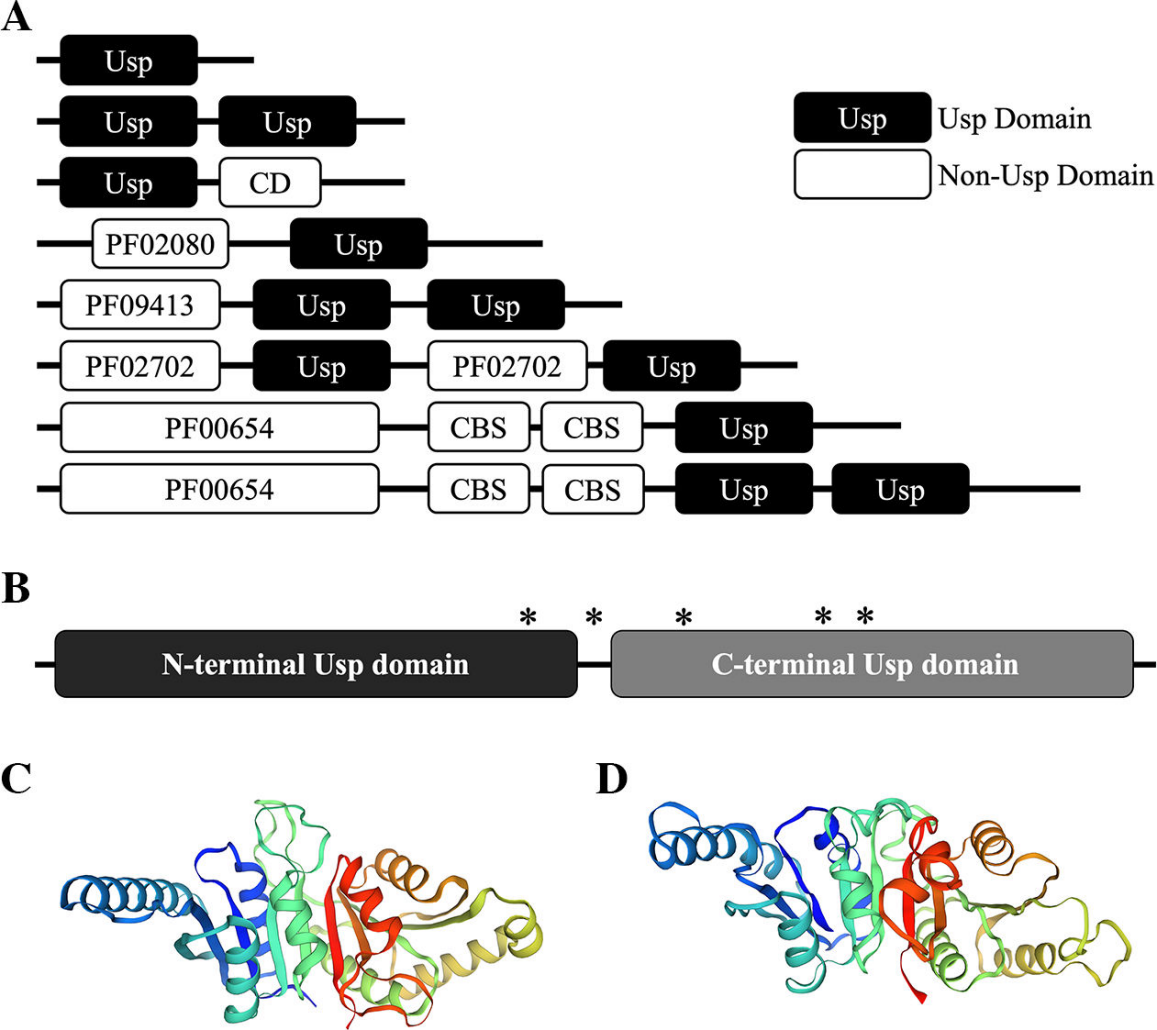
Usp domains in proteins from various bacteria and in *F. tularensis* Usp. (**A**) Illustration of commonly found bacterial proteins with one or two Usp domains (black) that are either without or with other non-Usp domains (white). In the bacterial proteins shown, non-usp domains included a conserved domain (CD) of unknown function, a cystathionine beta synthase (CBS) domain, and domains labeled with a Pfam family identifier. The domains with a Pfam identifier include PF02080 (a TrkA-C domain with unknown function), PF09413 (a DUF2007 domain in the Pii-type family of putative prokaryotic signal-transducing proteins), PF02702 (a histidine kinase sensor KdpD domain with putative osmoregulatory and potassium homeostasis functions), and PF00654 (a voltage-gated chloride channel domain). This information was obtained from a previously published review (32). (**B**) Diagram of the full-length 278 amino acid *F. tularensis* two-domain Usp with both the N- (black, residues 5–135) and C-terminal domains (gray, residues 152–275) each identified as “UspA” COG0589 by the NCBI Conserved Domain Tool. The asterisk (*) represents the approximate location of an amino acid difference in Usp between the *F. tularensis* subpopulations shown in Fig. S1. The *F. tularensis* SCHU S4 Usp structural model Q5NI44 shown in (**C**) was acquired using the SWISS-MODEL and AlphaFold three-dimensional prediction tools and based on (**D**) Usp (locus tag AF_1760) from *Archaeoglobus fulgidus* DSM 4304 crystal structure 3loq in the RCSB Protein Data Bank (PDB). Similar to *F. tularensis* Usp, *A. fulgidus* Usp (locus tag AF_1760) comprises two tandem Usp domains.

The Usp domain organization in bacteria has been previously shown to differ from one to two Usp domains that vary in position within certain proteins and is summarized in Fig. 1A (11, 32). *In silico* analysis of *F. tularensis* Usp indicated that this protein was 278 amino acids in length and contained two tandem Usp domains (Fig. 1B). Amino acids 5–135 (131 residues) comprising the N- and C-terminal Usp domains included amino acids 152–275 (124 residues). A sequence alignment of Usp from the different *F. tularensis* subpopulations showed that this protein shares over 98% amino acid identity, with only five clade-associated differences in residues 123, 144, 164, 202, and 205 (Fig. S2). Four of these amino acid differences were nonconservative, with one in the linker region and three in the C-terminal Usp domain (Fig. 1B; Fig. S2). Of note, residue 164 is a tyrosine in the hypervirulent A.I clade and a cysteine in the less virulent types A.II and B (Fig. S2). Since the oxidation of cysteine residues can alter protein structure and function (33), the residue 164 Usp polymorphism may contribute to additional stability of this protein during oxidative stress in hypervirulent A.I strains.

To verify that *F. tularensis* Usp contained two tandem Usp domains, the NCBI Conserved Domain Database Tool was used (17). These evaluations confirmed the presence of the two tandem Usp domains and also indicated that both of these domains belonged to the UspA family (accession number COG0589). To assess the similarity between these two domains, a sequence alignment was performed using the NCBI Global Alignment Tool with the Needleman–Wunsch algorithm. This comparison revealed that these two *F. tularensis* Usp domains shared 30 and 50% amino acid identity and similarity, respectively. These findings suggested a common ancestor and potential gene duplication due to the content similarity and tandem organization, but that each Usp domain evolved independently with most likely complementary functions.

Although the two tandem *F. tularensis* Usp domains belong to the UspA family accession number COG0589 and the universal stress protein family A superfamily with identifier IPR006015, these domains share only 20% amino acid identity to UspA in *E. coli* (Table 1). In *M. tuberculosis*, Usp classification is based on length and domain content (19). More specifically, Class I includes proteins that are approximately 150 amino acids in length and comprise only a single Usp domain protein; Class II contains proteins that are about 275 amino acids in length with one Usp domain and a non-Usp C-terminal domain; and Class III includes proteins that are 305 amino acids in length with two tandem Usp domains. In some bacterial species, Usp homologs have been categorized based on the ability of these proteins to bind ATP and/or cAMP via a Walker A-like binding motif GXXGX_9_G(S/T/N) (19, 20, 25, 34). In *F. tularensis*, however, neither the N- nor C-terminal Usp domains contain a canonical Walker A-like motif and lack a critical glycine at the fourth residue within this motif, similar to *Haemophilus influenzae* UspA (HI_0815) that does not bind ATP (Table S1) (20). In general, the Class III classification scheme used for *M. tuberculosis* Usp homologs appears to be the most amendable for the categorization of *F. tularensis* Usp.

**TABLE 1:** Comparison of universal stress protein (Usp) domains in *F. tularensis* Usp to other microbial Usp homologs

*F. tularensis* two-domain Usp (amino acid length)*^a^*	Microbial Usp with locus tag (Usp name and Usp domain location, if applicable)^*b*^	Total Usp paralogs in microbial species	Usp domain residues (total domain length)	Usp domain % amino acid identity	Usp domain % amino acid similarity
**N-terminal Usp domain (131 residues**)	***A. baumannii***	6			
	H0N28_10165		1–145 (145)	26%	42%
	***A*. *fulgidus***	7			
	AF_1760 (N-terminal domain)		1–139 (139)	31%	51%
	AF_1760 (C-terminal domain)		150–267 (118)	22%	39%
	***C. burnetii***	2			
	CBU_1916		4–143 (140)	29%	53%
	CBU_1983		3–142 (140)	35%	52%
	***E*. *coli***	6			
	b3495 (UspA)		1–144 (144)	20%	41%
	b1895 (UspC)		1–142 (142)	22%	43%
	b3923 UspD		1–142 (142)	21%	44%
	b1333 (UspE, N-terminal domain)		3–146 (144)	17%	34%
	b1333 (UspE, C-terminal domain)		157–299 (143)	27%	46%
	b1376 (UspF)		1–144 (144)	25%	48%
	b0607 (UspG)		1–142 (142)	28%	48%
	***H*. *influenzae***	1			
	HI_0815 (UspA)		2–141 (140)	22%	43%
	***M. jannaschii***	2			
	MJ0577		1–143 (143)	27%	46%
	***M. tuberculosis***	9			
	Rv2005c (N-terminal domain)		9–148 (140)	26%	47%
	Rv2005c (C-terminal domain)		161–293 (133)	26%	51%
	***P. aeruginosa***	3			
	PA3017		6–141 (136)	37%	49%
**C-terminal Usp domain (124 residues**)	***A. baumannii***	6			
	H0N28_10165		1–145 (145)	29%	51%
	***A*. *fulgidus***	7			
	AF_1760 (N-terminal domain)		1–139 (139)	27%	44%
	AF_1760 (C-terminal domain)		150–267 (118)	22%	39%
	***C. burnetii***	2			
	CBU_1916		4–143 (140)	26%	47%
	CBU_1983		3–142 (140)	27%	44%
	***E*. *coli***	6			
	b3495 (UspA)		1–144 (144)	20%	41%
	b1895 (UspC)		1–142 (142)	22%	43%
	b3923 UspD		1–142 (142)	21%	44%
	b1333 (UspE, N-terminal domain)		3–146 (144)	17%	34%
	b1333 (UspE, C-terminal domain)		157–299 (143)	27%	46%
	b1376 (UspF)		1–144 (144)	25%	48%
	b0607 (UspG)		1–142 (142)	27%	44%
	***H*. *influenzae***	1			
	HI_0815 (UspA)		2–141 (140)	22%	43%
	***M. jannaschii***	2			
	MJ0577		1–143 (143)	22%	41%
	***M. tuberculosis***	9			
	Rv2005c (N-terminal domain)		9–148 (140)	24%	45%
	Rv2005c (C-terminal domain)		161–293 (133)	27%	44%
	***P. aeruginosa***	3			
	PA3017		6–141 (136)	30%	43%

^*a*^ Francisella tularensis SCHU S4 NCBI Reference Sequence NC_006570.2 (locus tag FTT_0245) 278 residue Usp was used for the amino acid comparison of the N- (residues 5–135) and C-terminal Usp domains (residues 152–275). Usp domain(s) were identified using NCBI’s Conserved Domain Database and/or EMBL-EBI’s InterProScan resources. Percent amino acid identity and similarity between Usp domains were obtained using the NCBI Global Alignment Tool with the Needleman–Wunsch algorithm for the overall alignment of the respective two Usp domains. Microbial genomes used included *Acinetobacter baumannii* ATCC 17978 NCBI Reference Sequence NZ_CP059041.1, *Archaeoglobus fulgidus* DSM 4304 NCBI Reference Sequence NC_000917.1, *Coxiella burnetii* RSA 493 NCBI Reference Sequence NC_002971.4, *Escherichia coli* str. K-12 substr. MG1655 NCBI Reference Sequence NC_000913.3, *Haemophilus influenzae* Rd KW20 GenBank L42023.1, *Methanocaldococcus jannaschii* DSM 2661 NCBI Reference Sequence NC_000909.1, *Mycobacterium tuberculosis* H37Rv NCBI Reference Sequence NC_000962.3, and *Pseudomonas aeruginosa* PAO1 NCBI Reference Sequence NC_002516.2.

^*b*^ When applicable, each domain in proteins comprised two tandem Usp domains that were analyzed separately. For microbes encoding more than Usp paralog, only the Usp with the highest percent amino acid identity and similarity to *F. tularensis* Usp was included, with the exception of *C. burnetii* and *E. coli* in which all Usp paralogs are shown.

^*c*^ Bacterial species associated with the respective Usp homolog is denoted in bold.

Since protein structure, domain content, and the spatial location of specific residues may assist in deciphering function (35), the primary sequence of *F. tularensis* Usp was used to obtain a model of this protein based on a crystalized Usp that shared the highest overall amino acid similarity. The model Q5NI44 shown in Fig. 1C was derived for *F. tularensis* SCHU S4 Usp (locus tag FTT_0245) using SWISS-MODEL and AlphaFold three-dimensional prediction tools. This model was based on the crystal structure 3loq in the RCSB Protein Data Bank (PDB) for *Archaeoglobus fulgidus* DSM 4304 Usp (locus tag AF_1760, Fig. 1D), which comprises two tandem Usp domains and was recombinantly produced in an *E. coli* expression system (32). The N- and C-terminal Usp domains in this *A. fulgidus* protein (locus tag AF_1760) share between 39 and 51% amino acid similarity with the *F. tularensis* Usp domains (Table 1). These assessments revealed that all four nonsynonymous residues and all cysteines in Usp from the different *F. tularensis* clades were predicted to be on the outer surface, including the 164 tyrosine/cysteine polymorphism. However, the effect of these amino acid differences is currently unknown.

The linker region that separates the two *F. tularensis* Usp domains is 15 residues without any prolines and considered a medium length and infers the lack of stiffness (36). The presence of two tandem Usp domains in *F. tularensis* Usp, which differ by 30 and 50% in amino acid identity and similarity, respectively, suggests both unique and shared functions. Tandem repeated domains reveal unusual structural malleability in case of domain atrophy and are predicted to be more resistant to misfolding (37). In addition, tandemly repeated domains are one of the most abundant classes of protein–protein interaction domains and function in nearly every cellular process, including transcriptional regulation (38). These conserved structural features in Usp within all the *F. tularensis* clades most likely contribute to the fitness of this pathogen.

The Basic Local Alignment Search Tool (BLAST) was initially used to identify homologs with the highest similarity to each of the two domains in *F. tularensis* SCHU S4 Usp from unrelated microbial species. Next, the residues comprising the Usp domain(s) were identified using NCBI’s Conserved Domain Database and/or EMBL-EBI’s InterProScan resources, and percent amino acid identity and similarity between Usp domains were obtained using the NCBI Global Alignment tool. These assessments showed that Usp from gram-negative bacteria, acid-fast bacteria, and an archaeon microbe had greater than 33% amino acid similarity to one of the two *F. tularensis* Usp domains (Table 1). Usp domains in specific Usp paralogs from *Archaeoglobus fulgidus*, *Coxiella burnetii*, *M. tuberculosis*, and *Pseudomonas aeruginosa* shared the highest amino acid similarity to the N-terminal Usp domain from *F. tularensis*, which ranged from 49 to 53% similarity. The C-terminal Usp domain from *F. tularensis* shared the highest amino acid similarity to Usp from *Acinetobacter baumannii* locus tag H0N28_10165 and *E. coli* UspF locus tag b1376 with 51 and 48% similarity, respectively. The length of the annotated N- and C-terminal Usp domains from these homologs ranged from 118 to 145 residues, similar to the 131 and 124 residue N- and C-terminal Usp domains in *F. tularensis*, respectively (Table 1). For the two tandem Usp homologs evaluated, *M. tuberculosis* Usp locus tag Rv2005c had the highest overall similarity to the two Usp domains in *F. tularensis* Usp. This *M. tuberculosis* Usp paralog was shown to be transcriptionally upregulated during hypoxic conditions (39, 40). However, the *P. aeruginosa* single-domain Usp (locus tag PA3017) shared the highest amino acid identity to both the N- and C-terminal Usp domains in *F. tularensis*. The diversity in microbes containing Usp homologs shows the widespread occurrence and adaptation of this protein family.

### *F. tularensis* Usp transcript is abundant and stable

**Fig 2:**
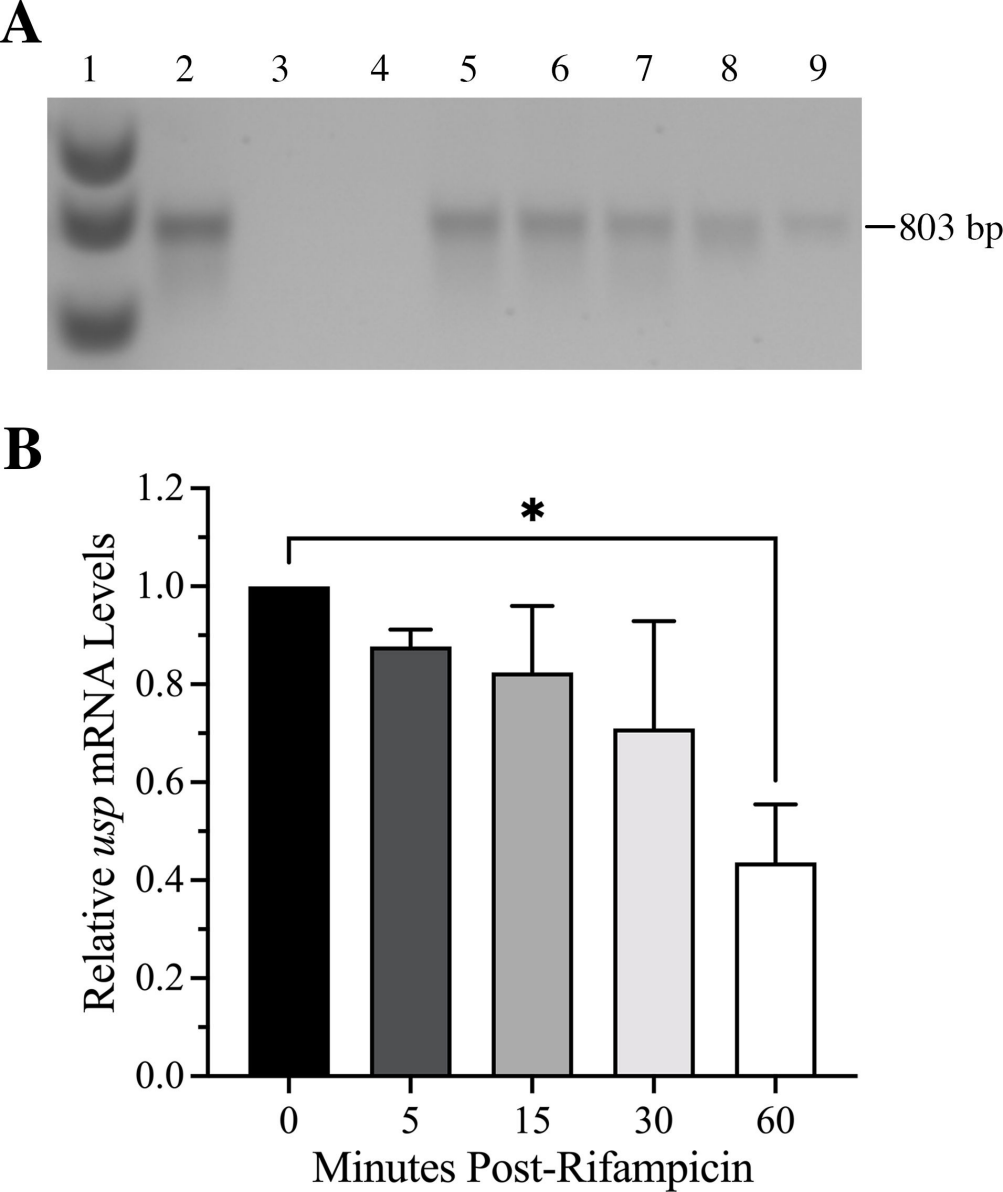
Assessment of *F. tularensis* Usp mRNA stability. (**A**) Representative gel with the RT-PCR products obtained for the no RTase control (lane 3), no template control (lane 4), and *F. tularensis* Usp transcripts during logarithmic growth before (lane 5) and after rifampicin treatment for 5, 15, 30, and 60 min (lanes 6, 7, 8, and 9, respectively). DNA size marker (lane 1) and the positive control for the 803 bp amplicon using *F. tularensis* genomic DNA as the template (lane 2) are also shown. (**B**) Graph representing *usp* transcript levels before and after rifampicin treatment using densitometry with ImageJ and normalization to untreated *F. tularensis* Usp mRNA. Data represent the mean ± standard error of the mean from two independent experiments. Statistical analysis was performed using an unpaired *T* test, and the asterisk (*) indicates that *usp* transcript levels were significantly reduced after 60 min of rifampicin treatment (*P* value < 0.05). Differences that were not significant are not shown (*P* value of > 0.05).

We previously determined that *usp* mRNA is monocistronic and maintained at high and unaltered levels throughout exponential growth and during the stationary phase in representative strains from the *F. tularensis* subpopulations (30). To determine if the *usp* promoter in the different *F. tularensis* subpopulations was conserved, a multiple sequence alignment of this intergenic region was assessed, and no nucleotide differences were observed (data not shown). Next, we evaluated the stability of the *usp* transcript during exponential growth using the RNA polymerase inhibitor rifampicin. The same volume of reverse transcription (RT)-PCR products obtained from the amplification of cDNA made with an equivalent amount of RNA from *F. tularensis* SCHU S4 without (0 min) and with rifampicin treatment after 5, 15, 30, and 60 min was gel-fractionated and stained for visualization (Fig. 2A). To quantify the resulting RT-PCR products, a densitometric analysis was performed using ImageJ, and the values were graphed (Fig. 2B). These data indicated that *usp* mRNA is stable with a half-life of over 30 min (Fig. 2), which is considerably longer than the average half-life of other bacterial transcripts that are typically only a few minutes (41).

**Fig 3:**
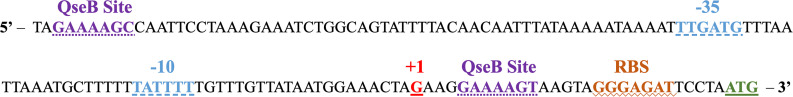
Promoter analysis of *F. tularensis* SCHU S4 *usp* and mapping of the *usp* transcriptional start site. 5′ RACE was used to identify the initial nucleotide in the 5′ UTR of the *usp* transcript (+1) and is shown in red and underlined. Several promoter analysis tools predicted the sigma factor RpoD (σ^70^) −35 and −10 binding regions that are shown in blue with a dashed underscore, as well as two putative transcription binding sites for the response regulator QseB, which are shown in purple with a dotted underline. The ribosomal binding site (RBS) is denoted in orange with a wavy underscore, and the translational start codon is in green and underlined.

To identify potential regulatory factors that control the transcription of *F. tularensis* Usp, the transcriptional start site was mapped using 5′ rapid amplification of cDNA ends (RACE). These experiments showed that the 5′ untranslated region (UTR) was 28 nucleotides in length and that the initial nucleotide to be transcribed was a guanine nucleotide (Fig. 3). The *F. tularensis* Usp promoter was then analyzed using several bacterial promoter analysis tools that identified the −35 and −10 regions, the Shine–Dalgarno ribosomal binding site, and several putative binding sites for the *F. tularensis* response regulator QseB (also known as PmrA) (Fig. 3). Others showed that the transcriptional expression of *usp* was reduced 6-fold in an *F. tularensis* Δ*qseB* mutant (42). Therefore, to evaluate the potential regulation of *usp* transcription by QseB, recombinant QseB with a C-terminal histidine tag (rQseB/His_6_) was produced, and the ability of this protein to bind to the *usp* promoter was evaluated using an electrophoretic mobility shift assay (EMSA). However, despite our numerous attempts to confirm the binding of QseB to the *usp* promoter, the results were inconclusive (Fig. S3), which may be due to the transient nature of this interaction and/or the need for additional factors.

To determine if stress factors would further enhance the already high levels of *F. tularensis* Usp mRNA, reverse transcription quantitative PCR (RT-qPCR) was performed with RNA isolated from mid-log phase SCHU S4 and LVS after exposure to acidic conditions, hydrogen peroxide, and the stringent response inducer serine hydroxamate (SHX). Late stationary phase RNA after 40 h of growth was also evaluated. *F. tularensis* strains were cultured in either brain heart infusion (BHI) broth or chemically defined medium (CDM), as described in the Materials and Methods section. Resulting threshold cycle (*C*_*t*_) values were normalized to the internal control and compared to the respective untreated strain in exponential growth. No substantial increase nor decrease in *usp* mRNA abundance was observed for any of these conditions (data not shown). Therefore, the elevated levels of *F. tularensis* Usp mRNA, along with the high stability of this transcript, may provide a fitness advantage by facilitating prompt translation of Usp when exposed to adverse surroundings.

### *F. tularensis* Usp levels are high and do not significantly change during various stress conditions

**Fig 4:**
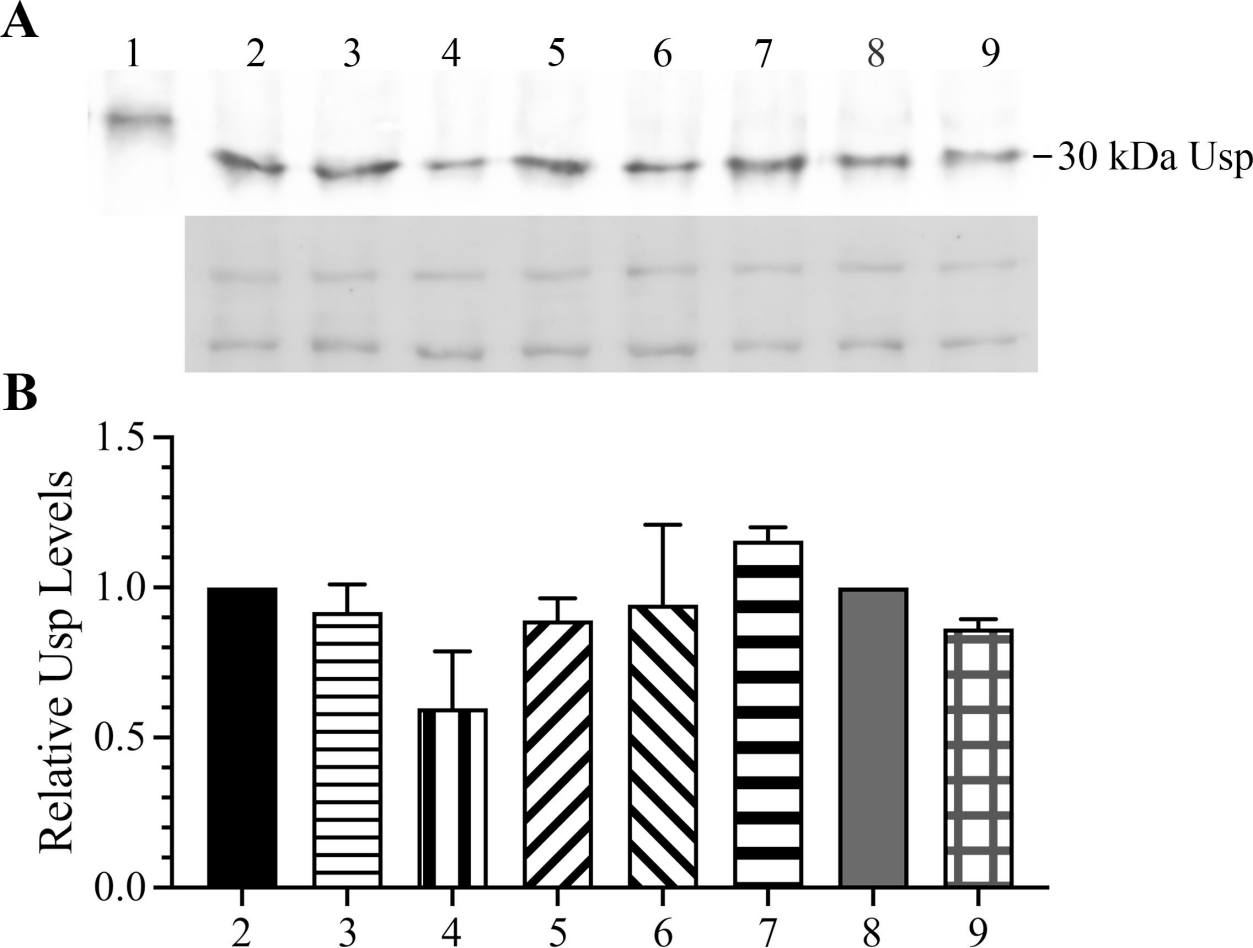
Immunoblot analysis of *F. tularensis* Usp abundance in the absence and presence of different stress conditions. (**A**) Shown in the top panel is a representative immunoblot of endogenous *F. tularensis* Usp levels without and with stress during growth and during the late stationary phase. The bottom panel shows the associated stained gel of two major proteins that were used to normalize the data. Recombinant Usp with a C-terminal histidine tag (0.2 µg) is shown as a positive control in lane 1, and endogenous Usp levels in an equivalent amount of the *F. tularensis* protein lysate was evaluated during growth in BHI without (lane 2) or with stress due to the presence of 1 and 5 mM hydrogen peroxide (lanes 3 and 4) or hydrochloric acid to acquire a low pH of 5.2 and 4.5 (lanes 5 and 6). Shown in lane 7 are the levels of Usp during the late stationary phase after 40 h of growth in BHI. *F. tularensis* Usp abundance was also evaluated in CDM without stress (lane 8) and with stress via amino acid depletion due to the addition of 10 µg/mL serine hydroxamate, a structural analogue of serine (lane 9). (**B**) Graph of endogenous *F. tularensis* Usp levels in the absence and presence of stress as described and shown in panel A. Relative raw integrated densities were normalized to the two associated major *F. tularensis* protein bands. Data represent the mean ± standard error of the mean from two independent experiments. A nonparametric unpaired *T* test was used and indicated that there was no significant difference in *F. tularensis* Usp levels without or with stress conditions (*P* value > 0.05).

A comparison of proteomes from representative *F. tularensis* A.I, A.II, and B strains was obtained from mass spectrophotometry analysis and revealed that the levels of *usp* were similar to the highly expressed lipoprotein LpnA (data not shown). We next sought to determine if there was a change in *F. tularensis* Usp levels when exposed to different stress factors. For these analyses, an anti-Usp polyclonal antibody was generated against denatured recombinant Usp with a C-terminal histidine tag (rUsp/His_6_). After the specificity of the primary antibody was confirmed to bind only *F. tularensis* Usp, the anti-Usp antibody was used to probe immunoblots with denatured lysates from *F. tularensis* that were either unstressed or stressed while in the mid-log growth phase. The stress conditions included treatment with 1 and 5 mM hydrogen peroxide, hydrochloric acid to obtain a final pH of 5.2 and 4.5, and SHX at 10 µg/mL. In addition, the levels of *usp* were assessed at the late stationary phase (40 h of growth). These immunoblot analyses showed that *F. tularensis* Usp levels were not substantially altered when exposed to these stress factors compared to untreated (Fig. 4). However, there was a slight but insignificant reduction of *usp* abundance when exposed to 5 mM hydrogen peroxide (Fig. 4). These data collectively demonstrated that neither the *F. tularensis* Usp transcript nor protein is induced by the stress conditions assessed, which is in contrast to other bacterial Usp homologs that have been evaluated (39, 43–45).

### *F. tularensis* Usp provides protection from superoxide stress

To determine a functional role for *F. tularensis* Usp, an in-frame markerless Δ*usp* mutant was constructed. PCR amplifications at this locus in WT and Δ*usp* produced the expected amplicon lengths, verifying the absence of the *usp* gene in the mutant (Fig. S4 and data not shown). In addition, southern blot and whole genome sequencing analyses with comparisons to WT confirmed that this gene was properly deleted and that no other chromosomal mutations or rearrangements occurred in Δ*usp*.

**Fig 5:**
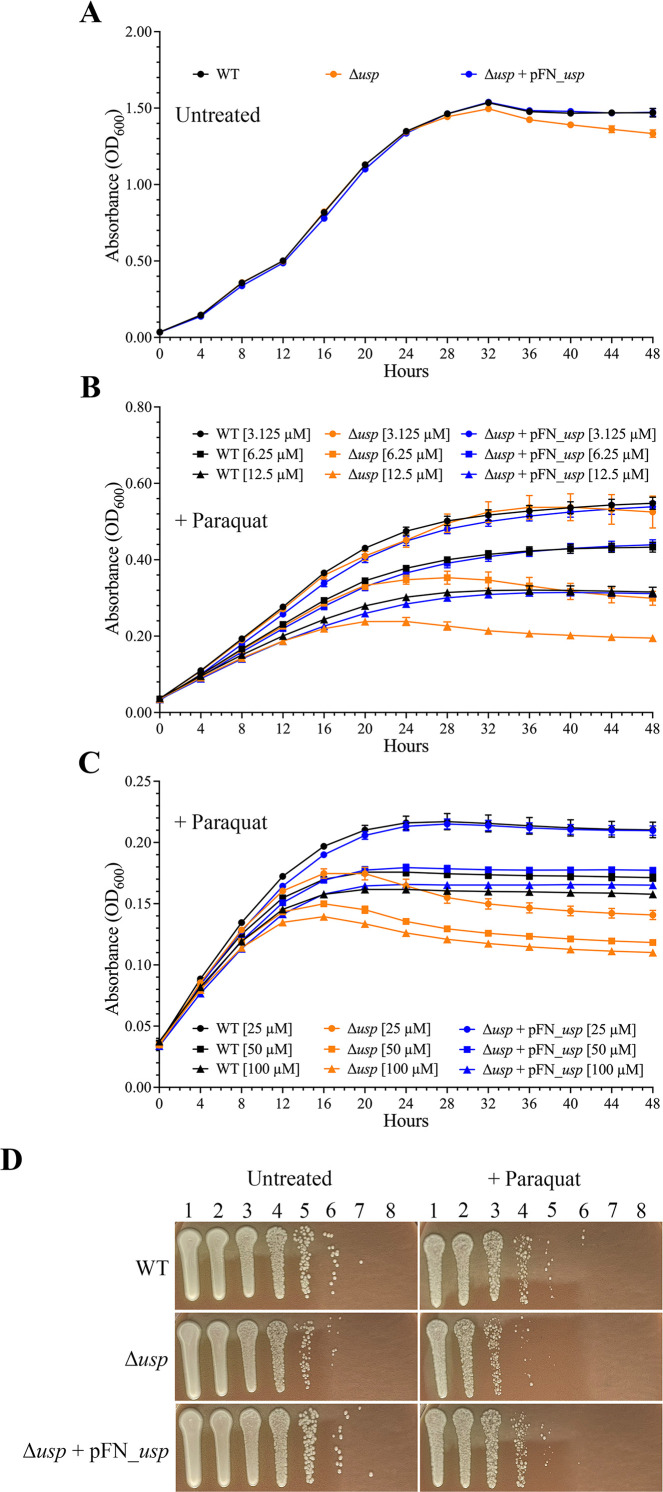
Growth comparison of *F. tularensis* WT, Δ*usp*, and transcomplemented Δ*usp* in the absence and presence of superoxide stress. The Usp expression plasmid pFN_*usp* was used to complement Δ*usp*, and paraquat was utilized to produce superoxide anions. Growth curves of the *F. tularensis* strains (**A**) without paraquat treatment, (**B**) with paraquat treatment at 3.125 (circle), 6.25 (square), and 12.5 µM (triangle), and (**C**) with paraquat treatment at 25 (circle), 50 (square), and 100 µM (triangle). Paraquat was added to the culture medium before the 37°C incubation for WT (black), Δ*usp* (orange), and transcomplemented Δ*usp* (blue). (**D**) Bacterial killing assay of *F. tularensis* WT, Δ*usp*, and transcomplemented Δ*usp* following treatment with 50 µM paraquat for 24 h. The serial diluted untreated (left panels) and paraquat treated (right panels) *F. tularensis* strains, which are denoted on the left after 4 days of incubation at 37°C are shown. Lane 1 contains undiluted *F. tularensis*, and lanes 2 through 8 contain 10^−1^, 10^−2^, 10^−3^, 10^−4^, 10^−5^, 10^−6^, and 10^−7^ serial diluted strains, respectively. Data shown represent at least two independent experiments.

The growth comparison of *F. tularensis* WT, Δ*usp*, and Δ*usp* containing the *usp* expression plasmid (complement strain) at 37°C in CDM without any treatment is shown in Fig. 5A. Initially, all three strains showed similar growth rates, but after 32 h, the Δ*usp* mutant began to lyse. Importantly, expression of *F. tularensis* Usp in the complement strain rescued the Δ*usp* mutant during the late stationary phase, preventing cell lysis (Fig. 5A).

Growth of *F. tularensis* Δ*usp* relative to WT was assessed in CDM with 1 to 10 mM hydrogen peroxide and in disc diffusion assays with 15 to 390 mM hydrogen peroxide and 8 to 215 mM cumen hydroperoxide. No growth difference was observed between these strains in any of these evaluations (data not shown). In addition, there was no difference in growth between *F. tularensis* Δ*usp* and WT in CDM with 400 mM sodium chloride, 0.125 to 0.75 µg/mL kanamycin, 1 and 10 µg/mL SHX, or acidic pH values of 5.2 and 4.5 (data not shown). However, in the presence of increasing concentrations of paraquat at 6.25 to 100 µM, *F. tularensis* Δ*usp* growth was substantially reduced in comparison to WT (Fig. 5B and C). At higher paraquat concentrations of 200 and 800 µM, the decrease in growth of Δ*usp* relative to WT was even more pronounced (data not shown). Importantly, Usp expression in the complement strain at each paraquat concentration evaluated rescued growth to WT levels (Fig. 5B and C). To further confirm the greater detrimental effect of paraquat on *F. tularensis* Δ*usp* relative to WT, a bacterial killing assay was performed. For these assessments, strain recovery after 24 h in CDM without and with 50 µM paraquat exposure was compared by measuring subsequent growth on chocolate agar plates with 0.4% glucose and 10 µg/mL kanamycin. These experiments showed that the complement strain expressing Usp recovered and grew to levels similar to WT, while Δ*usp* recovery and growth were reduced in both the absence and presence of paraquat (Fig. 5D). Together these data demonstrated that *F. tularensis* Usp serves a protective function that promotes survival in both the absence and presence of superoxide anions induced by paraquat.

### *F. tularensis* Usp levels remain elevated, even when exposed to high concentrations of paraquat for extended periods of time

**Fig 6:**
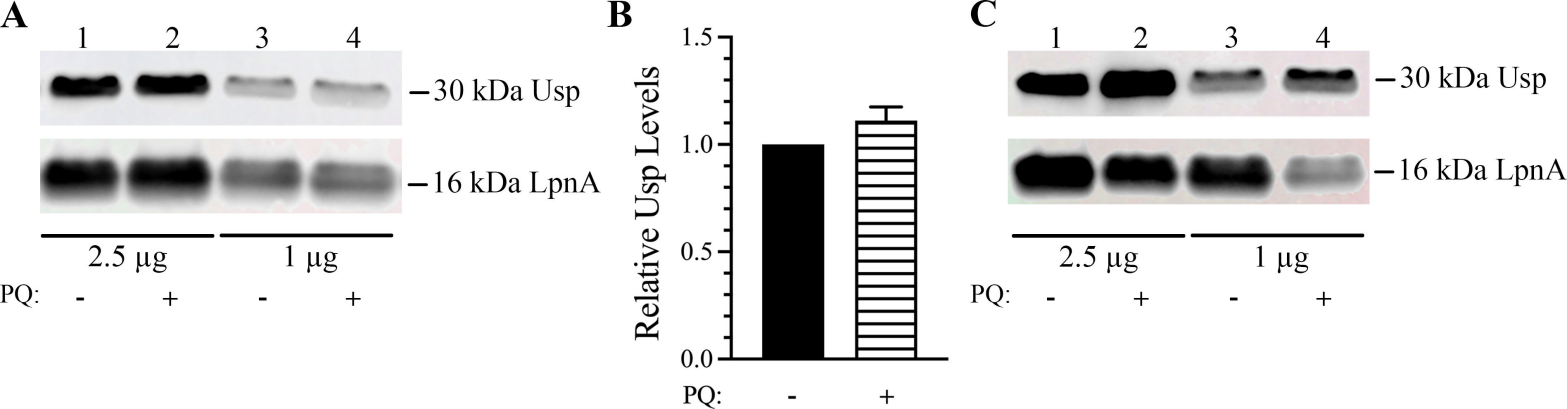
Immunoblot analysis of *F. tularensis* Usp levels during 50 µM and 0.5 mM paraquat stress. (**A**) Representative immunoblot showing no change in the abundance of *F. tularensis* 30 kDa Usp (top panel) and 16 kDa LpnA (bottom panel, internal control) with 50 µM paraquat treatment for 1 h (lanes 2 and 4) relative to untreated WT cultures (lanes 1 and 3) for 2.5 and 1 µg of protein lysate. (**B**) Graph of *F. tularensis* WT Usp levels without and with 50 µM paraquat treatment for 1 h from two biological replicates for 1 µg of protein lysate. Relative raw integrated densities were normalized to the associated *F. tularensis* LpnA internal control, and data represent the mean ± standard error of the mean. A nonparametric unpaired *T* test was used and indicated that there was no significant difference in *F. tularensis* WT Usp levels without or with 50 µM paraquat stress for 1 h (*P* value > 0.05). (**C**) Representative immunoblot showing an increase in *F. tularensis* 30 kDa Usp levels (top panel) and a decrease in 16 kDa LpnA levels (bottom panel) after treatment with 0.5 mM paraquat for 28 h (lanes 2 and 4) relative to untreated cultures (lanes 1 and 3) for 2.5 and 1 µg of protein lysate.

To determine if superoxide anions alter the abundance of *F. tularensis* Usp, immunoblots with lysates from untreated and paraquat treated *F. tularensis* WT were evaluated using anti-Usp specific antibodies. These data showed that there was no significant difference in *F. tularensis* Usp levels when untreated and treated with 50 µM paraquat for 1 h (Fig. 6A and B). However, when *F. tularensis* was subjected to a 10-fold higher concentration of paraquat (0.5 mM) for 28 h, Usp abundance increased approximately 1.4-fold (Fig. 6C). In contrast, exposure to 0.5 mM of paraquat for 28 h decreased LpnA abundance about 0.7-fold, indicating that this lipoprotein is not stable at high paraquat concentrations (Fig. 6C). These results notably demonstrated that Usp in *F. tularensis* WT remains abundant and intact, even in the presence of paraquat at high concentrations for an extended period of time.

### *F. tularensis* Usp transcript abundance remains high and unaltered by paraquat-induced superoxide stress

**Fig 7:**
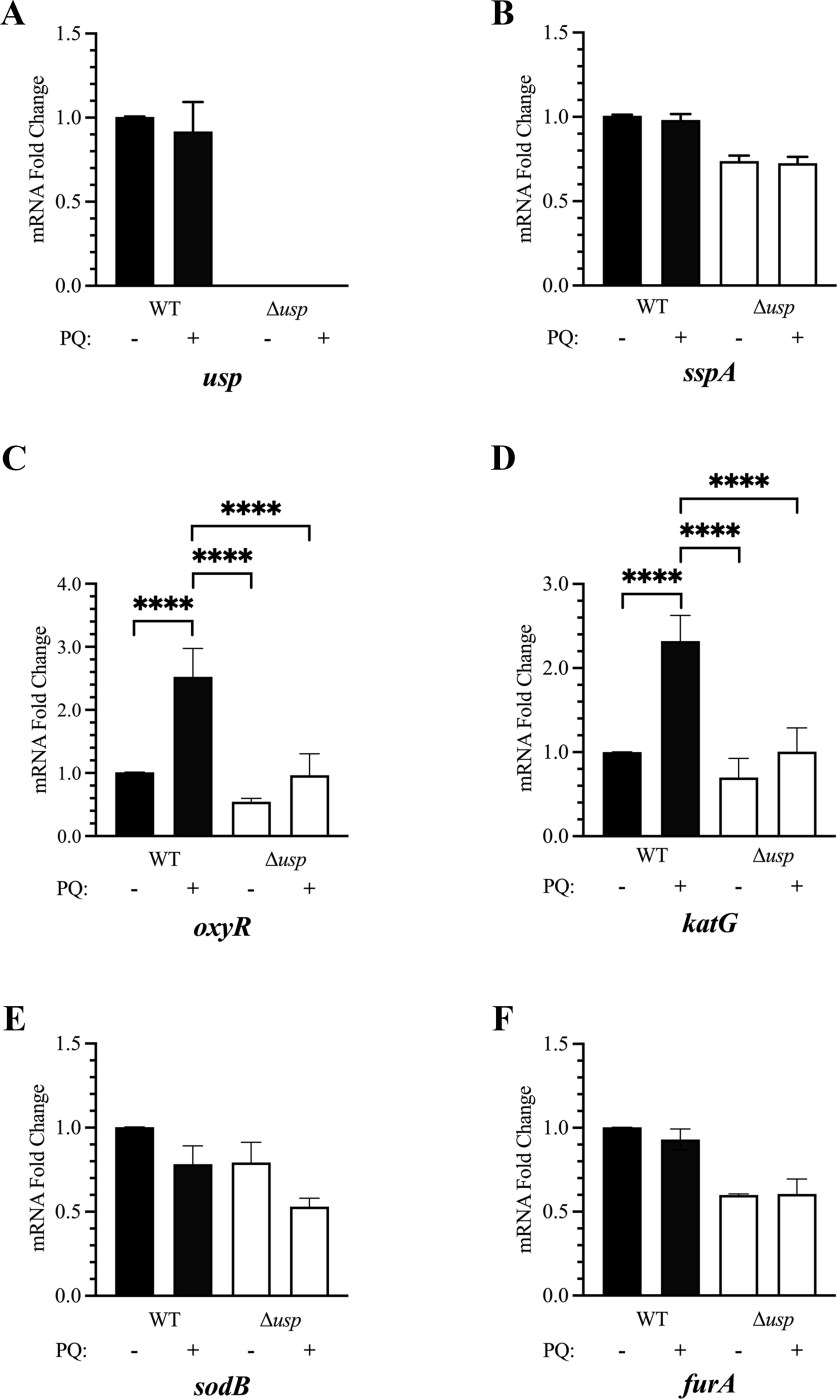
RT-qPCR analysis of regulatory, oxidative stress defense, and iron transport genes in *F. tularensis* WT and Δ*usp* in the absence and presence of superoxide stress. Shown are the relative changes in *F. tularensis* WT and Δ*usp* transcript levels for *usp* (FTL_0166), subsp. (FTL_1606), *oxyR* (FTL_1014c), *katG* (FTL_1504c), *sodB* (FTL_1791), and *furA* (FTL_1831) without or with superoxide stress induced by paraquat (PQ) treatment. *F. tularensis* strains were grown in CDM to mid-log and either untreated or treated with 50 µM paraquat for 1 h prior to RNA isolation. The data were normalized to the respective transcript in untreated WT, and the mean ± standard error of the mean from two or three biological experiments is shown. Two-way analysis of variance with Tukey’s post-hoc test was used, and only significant differences (*P* < 0.05) are shown. **** denotes a *P* value < 0.0001.

Since *F. tularensis* Usp promoted persistence in the presence of superoxide anions, transcriptional regulation of *usp* was assessed in the absence and presence of 50 µM paraquat for 1 h. When *usp*-specific primers were tested with cDNA derived from Δ*usp* RNA, no RT-qPCR products were obtained, further confirming the deletion of this gene (Fig. 7A). When WT was evaluated for changes in *usp* transcript abundance without and with paraquat treatment, no significant change was observed (Fig. 7A). In addition, the normalized C_t_ values (2^−ΔΔCt^) obtained for *F. tularensis* Usp mRNA were similar to the highly expressed *lpnA* transcript, verifying that the *usp* transcript was abundant. Collectively, these data revealed that *F. tularensis* Usp mRNA and protein levels in WT remain high during paraquat induced superoxide stress.

### *F. tularensis* Usp promotes an increase in *oxyR* and *katG* transcript levels during superoxide stress

To better understand the protective role of *F. tularensis* Usp in the presence of superoxide anions, the transcript levels of genes that encode major transcriptional regulators in *F. tularensis* were evaluated in the absence and presence of 50 µM paraquat for 1 h. These regulatory factors include the stringent starvation protein A subsp. (FTL_1606) and macrophage growth locus A MglA (FTL_1185). subsp. and MglA have been shown to heterodimerize and then bind to RNA polymerase, inducing the expression of many genes that promote *F. tularensis* survival (46). No change in subsp. mRNA abundance was observed in either WT and Δ*usp* when untreated and paraquat treated. However, subsp. transcript levels were 1.5-fold lower in the Δ*usp* mutant compared to WT, regardless of the absence and presence of paraquat (Fig. 7B). No difference in *mglA* transcript levels was observed between WT and Δ*usp* in the absence or presence of paraquat, with both strains exhibiting a slight reduction in the abundance of this mRNA when treated with paraquat (data not shown). These findings suggest a potential but minor role for Usp in maintaining the levels of subsp. mRNA, whereas *mglA* transcript abundance is independent of Usp, regardless of the absence or presence of superoxide anions.

Genes known to be involved in *F. tularensis* antioxidative defense were also assessed, including *oxyR* (FTL_1014 c), *katG* (FTL_1504 c), and *sodB* (FTL_1791). The transcriptional expression of both *oxyR* and *katG* notably increased over 2-fold in WT treated with paraquat compared to untreated, whereas only a minimal increase in these transcripts was observed in Δ*usp* during superoxide stress (Fig. 7C and D). Although no significant difference in *sodB* transcript levels was detected between WT and Δ*usp* without paraquat treatment, a modest decrease in *sodB* mRNA abundance was observed in the paraquat-treated mutant (Fig. 7E). Other antioxidative defense genes evaluated for transcript levels in *F. tularensis* WT and Δ*usp* during paraquat treatment included *ahpC* (FTL_1015), *sodC* (FTL_0380), *gpx* (FTL_1014c), and a Dyp-type gene (FTL_1773). There was no difference in the abundance of *ahpC* mRNA between WT and Δ*usp* in the absence or presence of paraquat. However, a 2-fold increase in *ahpC* transcript levels occurred during superoxide stress in both strains, indicating Usp-independent regulation of this gene. No other significant difference in transcript abundance for the other three antioxidant defense genes occurred in either WT or Δ*usp* without or with paraquat treatment. Therefore, since *F. tularensis oxyR* and *katG* encode crucial proteins that act cooperatively to protect against damage from reactive oxygen species (ROS) (47), Usp importantly contributes to the survival of this pathogen by enhancing the expression of these genes.

The toxic herbicide paraquat generates damaging free radicals, such as superoxide anions, peroxynitrite, and hydrogen peroxide (48). Of these, hydrogen peroxide can be further converted to destructive hydroxyl radicals via Fenton chemistry in the presence of an iron catalyst (48). *F. tularensis* possesses several iron acquisition systems and factors that regulate intracellular levels of this essential metal, which can be modulated to minimize oxidative damage to cellular components (49–51). To determine if transcript abundance for gene products involved in iron metabolism differed between *F. tularensis* WT and Δ*usp* in the absence or presence of superoxide anions, *furA* (FTL_1831), *fnr* (FTL_1302), *fslA* (FTL_1832), *feoA* (FTL_0660), and two *fdx* genes (FTL_1329 and FTL_0089) were evaluated. The levels of these iron associated transcripts were similar in WT or Δ*usp*, regardless of the absence or presence of paraquat with the exception of *furA*. In both the untreated and paraquat-treated mutants, the abundance of *fur* mRNA was approximately 1.6-fold lower (Fig. 7F). These data indicated that *F. tularensis* Usp contributes to modestly higher *furA* transcript levels that are independent of superoxide stress.

### Superoxide stress induced by paraquat decreases catalase activity

To determine if the higher *katG* transcript levels in *F. tularensis* WT versus the Δ*usp* resulted in a higher catalase activity, the lysates from these untreated or paraquat treated strains were assessed using two different methods. In-gel assays showed that catalase activity was slightly reduced in Δ*usp* compared to WT, regardless of the absence and presence of paraquat, and that paraquat treatment noticeably decreased the activity of this enzyme in both strains (Fig. S5A). To obtain more quantitative results, catalase activity in untreated and paraquat-treated WT and Δ*usp* was assessed using a spectrophotometer-based assay. These assays also showed that catalase activity was slightly lower in Δ*usp*, regardless of whether these strains were untreated or paraquat treated (Fig. S5B). Of note, there was almost a 2-fold decrease in catalase activity in both WT and Δ*usp* after paraquat treatment, confirming the detrimental effect of this toxic compound (Fig. S5B). These findings collectively support the role of *F. tularensis* Usp in counteracting the degradative effects of superoxide anions by increasing the *katG* transcript levels for subsequent catalase/peroxidase production.

## DISCUSSION

Although there are substantially more Usp entries for bacteria than for archaea and eukaryotes in the Pfam protein database, assigning function has remained a challenge since bacteria usually contain two or more Usp paralogs with overlapping roles (10). In contrast to the majority of bacteria, the small 1.9 Mbp genome of *F. tularensis* contains only one *usp* gene. However, *F. tularensis* Usp comprises two tandem Usp domains that differ by 30 and 50% in amino acid identity and similarity, respectively, allowing for both unique and shared functions. Since tandemly repeated domains are often involved in protein–protein interactions and transcriptional regulation (38), the tandem Usp domains in *F. tularensis* Usp may interact with each other, other proteins, and nucleic acids to regulate a variety of cellular processes.

We show that the half-life of the *usp* transcript was greater than 30 min (Fig. 2), which is considerably longer than most bacterial mRNA half-lives that are typically only a few minutes (41). For comparison, the *E. coli uspA* transcript has a half-life of only 1.8 min during steady-state growth (14). Furthermore, *F. tularensis* Usp transcript and protein levels remain high, regardless of the absence and presence of the stress conditions evaluated (Fig. 4, 6 and 7). These findings differ from the regulation of other bacterial Usp homologs, which have been shown to be induced by stress (39, 43–45). We also verified that *F. tularensis* Usp is unusually stable even when exposed to a high concentration of paraquat for an extended period of time. Comparisons of *F. tularensis* WT and Δ*usp* growth in the presence of the highly toxic compound paraquat showed that Usp serves a protective role, promoting persistence of this pathogen and contributing to enhanced expression of the antioxidant defense genes, *oxyR* and *katG*. However, whether Usp binds directly or indirectly to the *oxyR* and *katG* promoters for transcriptional upregulation or protects the associated gene products from degradation requires further study.

The ROS response in *F. tularensis* is made up of a complex network of regulatory genes that convert harmful redox species, such as superoxide anions, hydrogen peroxide, and hydroxyl radicals into less harmful, non-radical forms (52). In *F. tularensis*, OxyR upregulates the transcriptional expression of catalase/peroxidase KatG during oxidative stress by binding to the *katG* promoter but does not appear to have a role in iron metabolism (47, 52). In *E. coli*, the transcription factor OxyR has been shown to dimerize during oxidative stress due to disulfide bond formation between Cys199 and Cys208 (53). The *F. tularensis* OxyR transcription factor shares 36% overall identity in a 289-residue overlap with the 305 amino acid *E. coli* OxyR. *F. tularensis* OxyR also contains the Cys199 and Cys208 residues responsible for disulfide bond formation in *E. coli* OxyR. These shared features suggest that *F. tularensis* OxyR dimerizes during ROS stress for subsequent *katG* promoter binding and the upregulation of *katG* transcription. Moreover, the reduced expression of the critical oxidative defense genes *oxyR* and *katG* in *F. tularensis* Δ*usp* most likely contributed to the reduced ability of this mutant to persist during paraquat-induced superoxide stress relative to WT.

RNAseq data indicated that *usp* mRNA abundance decreased 0.46- and 0.57-fold without and with 1 mM hydrogen peroxide treatment, respectively, in a *F. tularensis* Δ*relA*/Δ*spoT* double mutant relative to WT (54). In a proteome study, Usp levels in an *F. tularensis* Δ*oxyR* mutant were unaltered compared to WT, and in the presence of 1 mM hydrogen peroxide, a 0.48-fold decrease in Usp levels in Δ*oxyR* was reported (52). These modest changes indicate that neither hydrogen peroxide nor these regulatory factors substantially affect *F. tularensis* Usp expression. Other investigations showed that *F. tularensis* Δ*sodB* is more susceptible to paraquat than WT (55). Our results showed that *sodB* mRNA levels did not significantly change in *F. tularensis* WT without or with paraquat treatment, while the abundance of this transcript was noticeably lower in paraquat-treated Δ*usp* relative to WT (Fig. 7E). These data suggest that Usp contributes to higher *sodB* mRNA levels by stabilizing this transcript during paraquat-induced superoxide stress.

**Fig 8:**
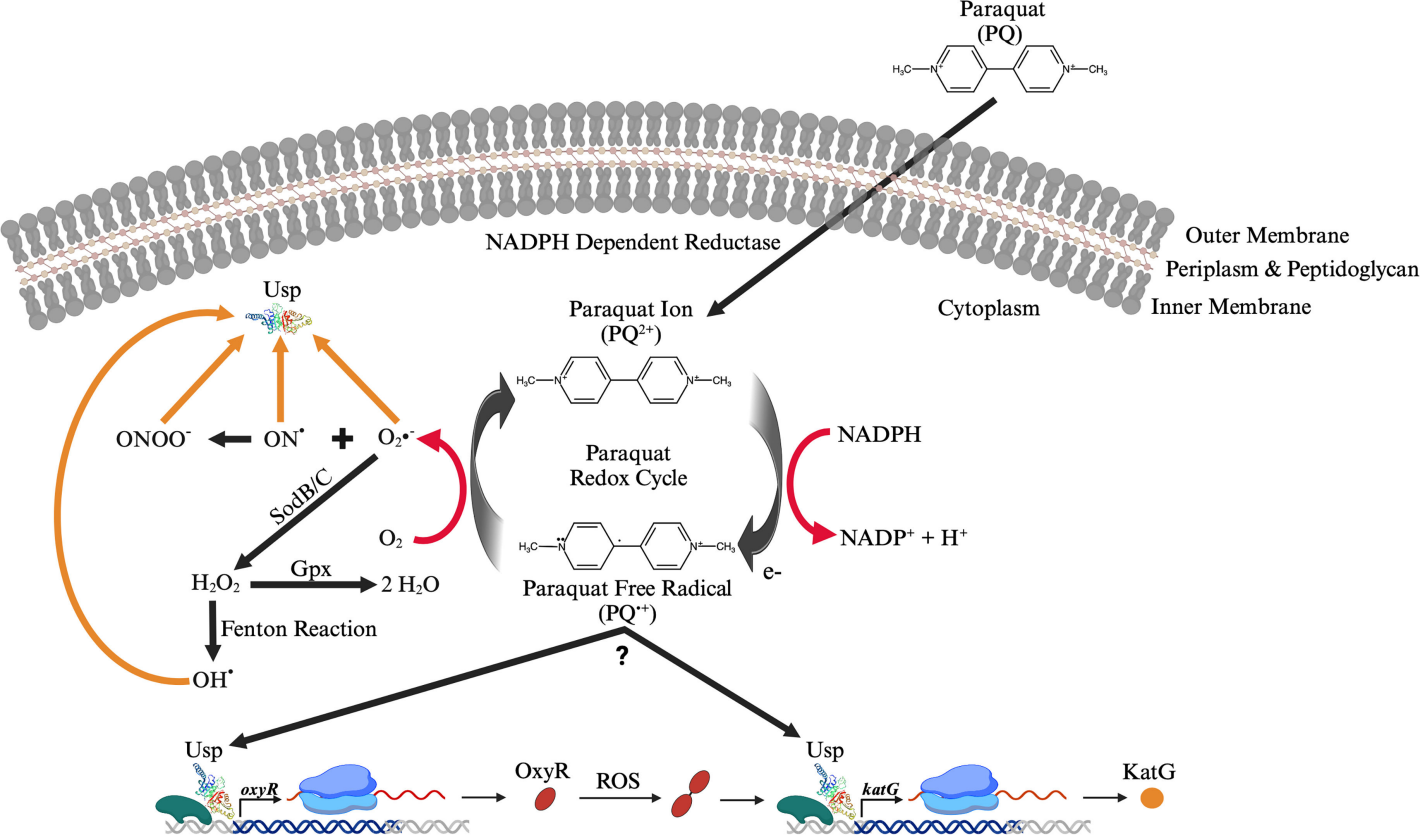
Working model of *F. tularensis* Usp protective functions during paraquat-derived superoxide stress. Uncharged paraquat (PQ) freely traverses the gram-negative cell wall of *F. tularensis*. Once in the cytoplasm, the PQ redox cycle is initiated by NADPH-dependent reductase that results in the generation of the PQ ree radical (PQ^•+^). PQ^•+^ then transfers an electron (e−) to oxygen (**O_2_**), generating a superoxide anion (O_2_^•−^). O_2_•^−^ reacts with nitric oxide (ON^•^) to generate peroxynitrite (ONOO^−^) or undergoes dismutation to produce hydrogen peroxide (H_2_O_2_) by superoxide dismutase (e.g., SodB and SodC). H_2_O_2_ reacts with an iron catalyst via the Fenton reaction to generate hydroxyl radicals (OH^•^). Glutathione peroxidase (Gpx) reduces H_2_O_2_ to water (H_2_O) and O_2_. Abundant *F. tularensis* Usp facilitates the transcriptional expression of the critical antioxidant genes *oxyR* and *katG* during superoxide stress and serves as a recipient of reactive oxygen species (ROS), preventing damage to other cytoplasmic and membrane components. Diagram was adapted from a PQ review by Blanco-Ayala et al. (48) and created with BioRender.com. Chemical structures were obtained by utilizing the Thermo Fisher Scientific Chemical Structure Search Tool.

Paraquat is one of the most widely used herbicides in the United States and only available to commercially licensed users according to the United States Environmental Protection Agency. This highly toxic compound has been banned in over 60 other countries (56). Paraquat acts as a source of superoxide anions via redox cycling (48). The reduction of this compound by NADPH-dependent reductase results in the consumption of NADPH and interferes with electron transport, both of which are required to maintain viability (48). The cofactor NADPH provides essential reducing equivalents that contribute to the regeneration of the ROS scavenger glutathione and is needed for both biosynthetic and anabolic reactions. The paraquat redox cycle generates superoxide anions that promote the production of hydrogen peroxide, hydroxyl radicals, and peroxynitrite (Fig. 8), all of which damage lipids, nucleic acids, proteins, and carbohydrates. We propose that maintaining high levels of *F. tularensis* Usp mRNA and protein allows this pathogen to promptly defend against ROS by promoting *oxyR* and *katG* expression and acting as a recipient of free radicals, reducing damage to other cellular components (Fig. 8).

Despite our numerous attempts to purify native *F. tularensis* Usp, this protein was consistently associated with genomic DNA and other cellular components. Global proteome analyses by others determined that *F. novicida* Usp is associated with both the membrane and the cytoplasm (57). Another global proteome study of *F. tularensis* subtype A.I and type B strains identified Usp in the membrane fraction (58), while bioinformatic analysis indicated that this protein was most likely cytosolic. These data collectively indicate that *F. tularensis* Usp associates with both nucleic acid and membrane components, supporting a protective role for Usp. Moreover, we previously determined that *F. tularensis* Usp is post-translationally modified with acetylated lysine and polyaminated glutamine residues, which may modulate binding to cellular components and affect function (27).

In *E. coli*, the genes encoding UspA, UspC, UspD, and UspE are induced upon entry into the stationary phase (14, 43). These four *E. coli* Usp paralogs have also been shown or are predicted to be regulated at the transcriptional level via a σ^70^-dependent promoter, which is coordinately regulated with other stationary genes by the alarmone guanosine tetraphosphate (ppGpp) (43, 59, 60). In another study, *E. coli* UspG was shown to be complexed with the heat shock protein GroEL during the stationary phase, prompting the authors to propose that the accumulation of this Usp paralog was due to growth inhibitory factors rather than starvation (61). Global transcriptome analysis of *M. tuberculosis* showed that several of the nine *usp* paralogs were transcriptionally induced upon entry into the stationary phase in well-aerated cultures and oxygen-depleted conditions (39), whereas quorum sensing was shown to positively regulate all 11 *Burkholderia glumae* Usp paralogs during the stationary phase (62). These differences in Usp regulation upon growth arrest between bacterial species and within the various Usp paralogs in a species imply an adaptive response to the associated niche that prolongs bacterial viability.

In the current study, we importantly observed higher persistence of *F. tularensis* WT relative to Δ*usp* after extended periods in the stationary phase, even in the absence of paraquat-induced superoxide stress. In the stationary phase, although metabolic activity is reprogrammed and reduced to preserve cellular energy, reactive byproducts and other factors accumulate that can be toxic and heighten oxidative stress. The reduced ability of the *F. tularensis* Δ*usp* mutant to persist during the late stationary phase may be due to the lower levels of *oxyR* and *katG* expression, making this strain more susceptible to oxidative damage. In addition, there are probably other critical factors and pathways that are negatively affected by the lack of Usp during the stationary phase, which reduce the ability of *F. tularensis* to persist. Perhaps the binding of Usp to the chromosome, membrane, and other proteins regulates *F. tularensis* metabolism, division, and other cellular activities, as well as protects macromolecules from ROS damage (Fig. 8). Nevertheless, *F. tularensis* must maintain a non-replicative dormant state when conditions are unfavorable in the environment and when in macrophages, the primary replication niche, this pathogen rapidly reproduces.

The findings presented in the current study demonstrate that *F. tularensis* Usp transcript and protein levels remain high, regardless of the absence or presence of stress factors, which differs from other bacterial Usp homologs studied to date. The consistently high levels and stability of *F. tularensis* Usp implicate that this conserved protein is readily available to protect against free radical damage while preserving viability until conditions are conducive for cellular division. *F. tularensis* is both a zoonotic and facultative intracellular pathogen, requiring adaptability to diverse surroundings. Unlike some intracellular pathogens that replicate in a membrane-bound vacuole within the infected host cell, *F. tularensis* replicates in the cytosol of infected macrophages, an innate immune cell that typically eliminates pathogens. During a macrophage infection, *F. tularensis* rapidly escapes the phagosome, induces autophagy, and then replicates to high numbers in approximately 24 h, prior to releasing a viable progeny that infects other host cells. In the environment, this pathogen encounters a variety of adverse and dynamic biotic and abiotic factors, including free radicals and nutrient-depleted conditions for extended periods of time. Although our preliminary results did not show a phenotypic difference between *F. tularensis* WT and Δ*usp* during the infection of a mouse macrophage cell line, the notable role of Usp in protecting against free radicals and promoting survival during growth arrest likely contributes to the ability of this zoonotic pathogen to persist in these environments. These capabilities further the transmission of the vector-borne disease tularemia and foster new enzootic reservoirs for *F. tularensis*. Acquiring a better understanding of the multifaceted mechanisms that *F. tularensis* utilizes to counteract oxidative stress, maintain redox homeostasis, and persist will undoubtedly enhance our knowledge on the biology and ecologic cycle of this Tier 1 select agent.

## MATERIALS AND METHODS

### Bacterial strains and culturing conditions

The *F. tularensis* strains used in this study included hypervirulent subtype A.I strain SCHU S4 and attenuated type B strain LVS. Both *F. tularensis* strains were obtained from Biodefense and Emerging Infections (BEI) Resources established by the National Institute of Allergy and Infectious Diseases (NIAID). Select agent *F. tularensis* strains were transferred to the University of Nebraska Medical Center in Omaha following the requirements of the Select Agent Program as outlined in the Animal and Plant Health Inspection Service/CDC Form 2, Guidance Document for Request to Transfer Select Agents and Toxin. Manipulation of viable culture material was performed by authorized individuals within a biosafety level 3 (BSL-3) laboratory certified for select agent work by the United States Department of Health and Human Services using laboratory biosafety criteria, according to requirements of the Federal Select Agent Program.

For each experiment, *F. tularensis* strains were cultured from a master stock onto chocolate agar plates (Remel) and incubated at 37°C for 2 days before further subculturing or processing. *F. tularensis* growth curves were obtained at 37°C with shaking using Chamberlain’s CDM that was prepared as previously described (63) or BHI broth supplemented with 0.2% cysteine, 1% hemoglobin, and 0.4% glucose without or with 10 µg/mL kanamycin as appropriate. Supplements added to CDM and BHI were obtained from Sigma-Aldrich (St. Louis, MO). For the cloning of the recombinant proteins, *E. coli* DH5α or TOP10F′ (New England Biolabs) was used and subcultured in Luria–Bertani (LB) containing 30 µg/mL kanamycin for selection. For the production of the recombinant proteins, clones were transformed into *E. coli* BL21 DE3 (Novagen) and subcultured in 2× yeast extract tryptone culture medium (2YT) broth. Growth curves were obtained utilizing a Tecan Spark microplate reader and 96-well plates, in which OD_600_ readings were recorded every hour or flasks when downstream sample processing was needed. All media used for experimentation were inoculated to a starting absorbance at 600 nm (OD_600_) of 0.01, 0.03, or 0.05.

### DNA manipulations and reagents

All oligonucleotide primers used in this study were synthesized by Thermo Fisher Scientific and listed in Table S2. Genomic DNA was isolated using cetyl trimethylammonium bromide (CTAB) according to standard protocols. Plasmid DNA was prepared with Zymoclean Gel DNA Recovery Kit (Zymo Research Corporation), as recommended by the manufacturer. DNA restriction enzyme digests, cloning, and electrophoresis were performed according to standard protocols. PCR was performed using Platinum Taq DNA polymerase high-fidelity enzyme and associated components (Invitrogen). Ligations were performed using a T4 DNA ligase (Roche) or NEBuilder HiFi DNA Assembly Master Mix (New England Biolabs). DNA fragments were purified using either a QIAquick PCR Purification or QIAquick Gel Extraction Kit (Qiagen). DNA concentrations were obtained using a NanoDrop spectrophotometer. DNA sequencing was performed by the University of Nebraska Medical Center Genomics Core Facility using Sanger sequencing. Plasmids were electroporated into *E. coli* and *F. tularensis* using 2.5 kV, 50 µF, and 125 ohms or 2.5 kv, 25 µF, and 600 ohms, respectively. An ECM 630 Electro Cell Manipulator (BTX Harvard Apparatus) with a 0.2 cm-gap cuvette was utilized for all electroporation procedures.

### Construction and complementation of *F. tularensis* Usp deletion mutants

*F. tularensis* LVS Δ*usp* was constructed via triparental conjugation, as previously described (64). Protocols pertaining to the construction and transcomplementation of this mutant are provided in our previous study (65). Oligonucleotides used for the construction of the Δ*usp* mutant and for the transcomplementation plasmid are provided in Table S2.

Deletion of *usp* in the resulting *F. tularensis* LVS Δ*usp* mutant was confirmed by PCR amplification of the relevant chromosomal locus and subsequent fractionation in an agarose gel and staining for comparison to the associated wild-type strain amplicon derived with the same primer pair. Content of this chromosomal locus in the *F. tularensis* LVS Δ*usp* mutant was verified by DNA sequencing in both directions. Whole genome sequencing, RT-qPCR, and immunoblot analysis further confirmed that *usp* was deleted in the Δ*usp* mutant.

For complementation of *usp*, PCR amplification of the *usp* gene along with 90 bp promoter region was performed. The resulting amplicon was then digested with *Kpn*I and *Bam*HI for subsequent cloning into these restriction endonuclease sites in pFNLTP6. This shuttle plasmid was constructed for stable maintenance in *F. tularensis*, as previously described (66, 67). The Usp expression plasmid was sequenced in both directions to confirm content. Expression of *usp* in the complemented Δ*usp* mutant was confirmed by RT-qPCR and immunoblot analysis.

### RNA processing and reverse transcription quantitative PCR

RNA was isolated using TRIzol according to the manufacturer’s recommendations and then DNase treated using Baseline-ZERO DNase enzyme and buffer (Lucigen) for 1 h at 37°C, followed by an additional DNase spike and incubation for 30 min. DNase-treated RNA was then purified using the Zymo Research RNA Clean & Concentrator-5 Kit (Zymo) as recommended by the manufacturer. RNA concentration was determined using the NanoDrop spectrophotometer, and RNA integrity was confirmed by fractionation in an agarose gel with subsequent staining.

First-strand cDNA synthesis was accomplished using SuperScript IV Reverse Transcriptase (Invitrogen), 0.1 µM gene-specific primers, and 1 µg RNA, as recommended by the manufacturer. RT-qPCR was performed with consistent dilutions of cDNA, PowerUp SYBR Green Master Mix, and the standard cycling mode for primers with a Tm of <60°C in a 7500 Fast DX Real Time PCR System (Applied Biosystems). The Sequence Detection Software version 1.4.1 (Applied Biosystems) was used for analysis of the RT-qPCR data. Relative quantification for the gene of interest to the internal control *lpnA* was obtained using the 2^−ΔΔCt^ equation, as previously described (68).

### 5′ Rapid Amplification of cDNA End (RACE)

The *F. tularensis* Usp transcriptional start site was mapped using the 5′/3′ RACE 2nd Generation Kit (Roche) following the manufacturer’s recommendations. As previously detailed, 2 µg of DNase-treated *F. tularensis* RNA and the 5′ RACE primers shown in Table S2 were used (30). The resulting amplicon was visualized by fractionation in a 1% agarose gel with subsequent staining, and sequencing was performed with the nested 5′ RACE primer (Table S2).

### RNA stability assay

To determine the half-life of *F. tularensis* Usp mRNA, SCHU S4 was grown in BHI broth to the mid-log growth phase. Just prior to the experiment, rifampicin (Sigma) was dissolved in dimethyl sulfoxide to obtain a final concentration of 100 mg/mL for the working stock and then stored on ice protected from light. Before adding rifampicin, 2 × 10^8^ cells were transferred to two volumes of RNAprotect Bacteria Reagent (Qiagen) and immediately vortexed for the 0 min RNA samples. Next, rifampicin was added to the remaining mid-log culture to obtain a final concentration of 0.5 mg/mL. After 5, 15, 30, and 60 min, 2 × 10^8^ cells were transferred to two volumes of RNAprotect Bacteria Reagent (Qiagen) and immediately vortexed. As a control, the same volume of dimethyl sulfoxide was added to a mid-log culture and processed as described for the other samples. All samples were incubated at room temperature and centrifuged, and then supernatants were discarded, as described in the RNAprotect Bacteria Reagent protocol.

RNA was isolated from the pelleted cells using TRIzol according to the manufacturer’s recommendation and then prepared for first-strand cDNA synthesis as described above, with the exception of the primer used. For these analyses, the *F. tularensis* Usp-specific reverse primer that would produce the full-length 803 bp RT-PCR product was used to reflect stability of the entire *usp* transcript. For RT-PCR amplification of full-length *usp* cDNA, high-fidelity Platinum Taq DNA polymerase (Invitrogen) was used with equivalent amounts of cDNA for each time point and the controls. Resulting amplicons were fractionated in a 1.2% agarose gel and stained for visualization, and then densitometric analysis was performed using ImageJ for quantitation. The area ratio for each cDNA post-rifampicin was normalized to the untreated 0 min cDNA and graphed using the normalized values with GraphPad Prism software.

### Production of recombinant Usp and QseB proteins

Recombinant proteins rUsp/His_6_ and rQseB/His_6_ were constructed following standard procedures with the oligonucleotides shown in Table S2. The amplicons were purified using the Roche High Pure PCR Product Purification Kit (Roche), digested with NcoI and XhoI, and cloned into the pET28a expression plasmid, which was digested with the same restriction endonucleases. PCR amplification of the cloned insert produced the expected size and Sanger sequencing by the University of Nebraska Medical Center Genomics Core Facility confirmed content.

For the expression of the recombinant rUsp/His_6_ and rQseB/His_6_ proteins, plasmids were electroporated into *E. coli* BL21(DE3) and induced with 0.5 mM isopropyl β-D-thiogalactopyranoside (IPTG) for 20 h at 18℃ with shaking in 2YT media. After induction, cultures were centrifuged, and cell pellets were resuspended in 45 mL lysis buffer containing 20 mM sodium phosphate dibasic, 500 mM sodium chloride, 30 mM imidazole, 1 µL/g aprotinin, and 1 mM phenylmethylsulfonyl fluoride (PMSF). Cells were then lysed via sonication for 10 s at 50% amplitude, which was repeated as needed while keeping the samples cold. Sonicated samples were either centrifuged to pellet insoluble cellular components or filtered using progressively decreasing pore sizes as appropriate to clarify supernatants.

Clarified supernatants containing rUsp/His_6_ and rQseB/His_6_ were purified using nickel–nitrilotriacetic acid (Ni–NTA) column affinity purification. After the Ni–NTA columns were equilibrated with 30 mM sodium phosphate dibasic, 500 mM sodium chloride, and 30 mM imidazole, supernatants were added. Columns were then washed with binding buffer, and rUsp/His_6_ and rQseB/His_6_ were eluted with 0.5 M imidazole. Eluted fractions were evaluated by 10% SDS-PAGE to confirm which fractions contained the protein of interest, and then fractions containing rUsp/His_6_ and rQseB/His_6_ were dialyzed against phosphate buffered saline (pH 7.4) or ammonium hydroxide (pH 11), respectively.

### Electrophoretic mobility shift assay

Purified *F. tularensis* rQseB/His_6_ at 0.5, 0.75, and 1.0 µg were evaluated with 500 ng of the 1000 bp *usp* promoter region in EMSA analyses, which were performed as was previously described (69). Bovine serum albumin (BSA) at 1 µg and 500 ng of the 458 bp *qseB* promoter region was used as negative and positive controls, respectively. *F. tularensis* Usp and *qseB* promoter regions were amplified using PCR and the primers shown in Table S2 and then purified. The relevant protein and DNA were combined and then mixed with binding buffer (20 mM Tris–HCl pH 8, 0.1 mM EDTA–Na_2_, 50 mM KCl, 10 µg/mL BSA, 5% glycerol, 20 mM MgCl_2_, 0.1 mM DTT, and 0.05% PEG 8000). Samples were incubated at either room temperature or on ice for 30 min. The entire sample was loaded into a 0.5% 0.33× TBE agarose gel that was run for 5 h at 50 volts and 4°C in 0.33× TBE buffer and then stained in 0.5 µg/mL ethidium bromide. Results were visualized using the Azure Biosystems c600 instrument with the UV302 autoexposure setting.

### Immunoblot analysis

The generation of polyclonal antibodies to denatured *F. tularensis* rUsp/His_6_ and immunoblot analysis of endogenous *F. tularensis* Usp was accomplished, as previously described (27). Immunoblots with lysates from WT and the Δ*usp* mutant confirmed the specificity of the primary antibody to only *F. tularensis* Usp and verified the absence of Usp in the mutant. Affinity-purified rUsp/His_6_ (0.2 µg) was included as a control. Briefly, initial immunoblots of *F. tularensis* proteins were separated on 10% SDS polyacrylamide gels, with each lane containing protein from equivalent numbers of cells. For these analyses, *F. tularensis* was either untreated or stressed with 1 or 5 mM hydrogen peroxide, hydrochloric acid acidified medium to an initial pH of 5.2 or 4.5, and 10 µg/mL DL-serine hydroxamate, a serine analog that inhibits seryl-tRNA synthetase. For the growth medium, BHI was used for these evaluations, except for serine starvation, in which CDM was used, and serine was replaced with DL-serine hydroxamate. For untreated *F. tularensis*, BHI or CDM was used for growth as appropriate. After transferring proteins to a 0.45 µm polyvinylidene fluoride Immobilon-P transfer membrane (Millipore), the membrane was stained with TotalStain Q (Azure Biosystems), and images were acquired on the Azure Biosystems c600 Imaging System using the Cy3 setting, as recommended by the manufacturer. To detect Usp, the membrane was rinsed with water and blocked with 5% dry milk in 1× Tris-buffered saline plus 0.1% Tween 20 (TBST) prior to immunodetection with a 1:4,000 dilution of the rabbit serum containing anti-Usp antibodies. After overnight incubation at 4°C, the membrane was rinsed twice with 1× TBST and then incubated with a 1:5,000 dilution of the AzureSpectra 700 goat anti-rabbit IgG antibody S1017 (Azure Biosystems) for 1 h. Next, the membrane was washed with TBST, followed by a wash with TBS that does not contain Tween 20. After the blot was dry, images were acquired using the Azure Biosystems c600 System and then imported into ImageJ for densitometric analysis. The raw integrated densities of the two major *F. tularensis* proteins in the total protein-stained blot were first summed together. Next, the raw integrated densities obtained for *F. tularensis* Usp with the treated conditions and the two major *F. tularensis* proteins were normalized to their respective untreated control. The normalized Usp densities were then divided by the normalized densities obtained for the two major proteins. The resulting values represent *F. tularensis* Usp levels relative to *F. tularensis* protein levels.

For immunoblot analysis of unstressed and paraquat stressed *F. tularensis,* the WT, Δ*usp* mutant, and transcomplemented strain were subjected to 50 µM paraquat treatment for 1 h after 22 h of growth in CDM containing 10 µg/mL kanamycin. *F. tularensis* WT was also cultured in CDM containing 10 µg/mL kanamycin for 12 h and then exposed to 0.5 mM paraquat for 28 h before processing for immunoblot analysis. These cultures were lysed by sonication in 10 mL of 50 mM potassium phosphate (pH 7.8), and protein concentrations were determined using the Bicinchoninic Acid (BCA) Protein Assay Kit (Pierce). Protein lysates were loaded onto 12% precast SDS gels (Bio-Rad) at protein concentrations of 2.5 and 1 µg. Protein transfer of *F. tularensis* Usp to polyvinylidene fluoride Immobilon-P was performed as described above. These immunoblots were blocked for 30 min in TBS with 5% dry milk and then hybridized with 10 µL (7 µg) of affinity-purified rabbit anti-denatured Usp polyclonal in 20 mL TBS with 5% dry milk. After overnight incubation at 4°C, blots were washed with TBST. For the secondary antibody, 2 µL (0.4 mg/mL in 50% glycerol) donkey anti-rabbit IgG conjugated to horse radish peroxidase (Jackson ImmunoResearch) was diluted into 20 mL TBS with 5% dry milk for subsequent incubation with the blot. After washing the blots with TBST, detection was performed with enhanced chemiluminescence (ECL) reagents Radiance Plus (Azure Biosystems), and images were acquired on the Azure Bioimaging c600 System. Next, immunoblots were washed with TBST and then hybridized with 2 µL of the mouse monoclonal anti-*F*. *tularensis* LpnA (BEI Resources, clone 164.75) that was diluted in 10 mL TBS with 5% dry milk. After washing the blots with TBST, an anti-mouse IgG conjugated to horse radish peroxidase (Cell Signaling) was used as the secondary antibody. After washing blots with TBST, detection was again performed with the ECL reagent, and images were acquired on the Azure Bioimaging c600 System. Immunoblot analyses were performed in at least two independent experiments and two biological replicates.

### Catalase assays

*F. tularensis* WT and Δ*usp* catalase activity was evaluated using protocols previously described using either a native gel stained for catalase activity (70) or a spectrophotometer-based catalase activity assay (71).

### Bioinformatic analysis

*F. tularensis* subsp. *tularensis* SCHU S4 NCBI accession file NC_006570.2 locus tag FTT_0245, *F. tularensis* subsp. *tularensis* WY96-3418 accession file NC_009257.1 locus tag FTW_1846, and *F. tularensis* subsp. *holarctica* LVS accession file NC_007880.1 locus tag FTL_0166 were used for the bioinformatic analyses. Microbial Usp domain organization and residues within the Usp-like domains were determined using NCBI’s Conserved Domain Database and/or the European Molecular Biology Laboratory–European Bioinformatics Institute’s (EMBL–EBI’s) InterProScan resources (17, 72). Multiple sequence alignments of genes, promoter regions, and proteins were acquired via Clustal Omega (73). The structural model for *F. tularensis* Usp was made by inputting the SCHU S4 Usp amino acid sequence into the three-dimensional prediction tools, SWISS-MODEL and AlphaFold, using the “Build Model” setting (74). Comparison of *F. tularensis* Usp nucleotide and amino acid sequence identity and similarity to other microbial Usp homologs was initially attained by utilizing BLAST (75). For overall percent amino acid identity and similarity between Usp domains, the NCBI Global Alignment tool with the Needleman–Wunsch algorithm for the overall alignment of proteins or specific domains within proteins was used. To identify *cis* and *trans* regulatory components in the *F. tularensis* Usp promoter, BPROM (76) and Virtual Footprint (77) were utilized.

### Statistical analysis

Data are presented as means ± standard error of the mean and representative of at least two biological replicates. Data from experiments with one variable were analyzed using Student’s *t*-test. Data from experiments with multiple variables were analyzed utilizing two-way analysis of variance and Tukey’s post-hoc test. Statistical significance was set at a *P* value of <0.05, and statistical analyses were performed using GraphPad Prism.

## SUPPLEMENTAL MATERIAL

Supplemental figures and tables jb.00377-24-s0001.pdf Fig. S1 to S5; Tables S1 and S2.

ASM does not own the copyrights to Supplemental Material that may be linked to, or accessed through, an article. The authors have granted ASM a non-exclusive, world-wide license to publish the Supplemental Material files. Please contact the corresponding author directly for reuse.
