# Supplementary material for: Francisella tularensis universal stress protein contributes to persistence during growth arrest and paraquat-induced superoxide stress
Source: J Bacteriol. 2025 Jan 23;207(2):e00377-24. doi: 10.1128/jb.00377-24 (PMC11841066; doi:10.1128/jb.00377-24)
Supplement: Supplemental figures and tables — Fig. S1 to S5; Tables S1 and S2. [file jb.00377-24-s0001.pdf]

Francisella tularensis Universal Stress Protein Contributes to Persistence During Growth Arrest and Paraquat-Induced Superoxide Stress

Benjamin Girardo, Yinshi Yue, Oksana Lockridge, Amanda M. Bartling, Lawrence M. Schopfer, Leonardo Augusto, and Marilynn A. Larson

SUPPLEMENTAL DATA

FIG S1 Genomic organization of the highly conserved gene encoding the universal stress protein (Usp) along with flanking genes. The single *usp* gene in representative strains from the different *F. tularensis* subpopulations (subtype A.I SCHU S4, subtype A.II WY96-3418, and type B LVS) along with flanking genes are shown. The conserved *usp* gene in each clade is shown in black and is enclosed with a black rectangle.

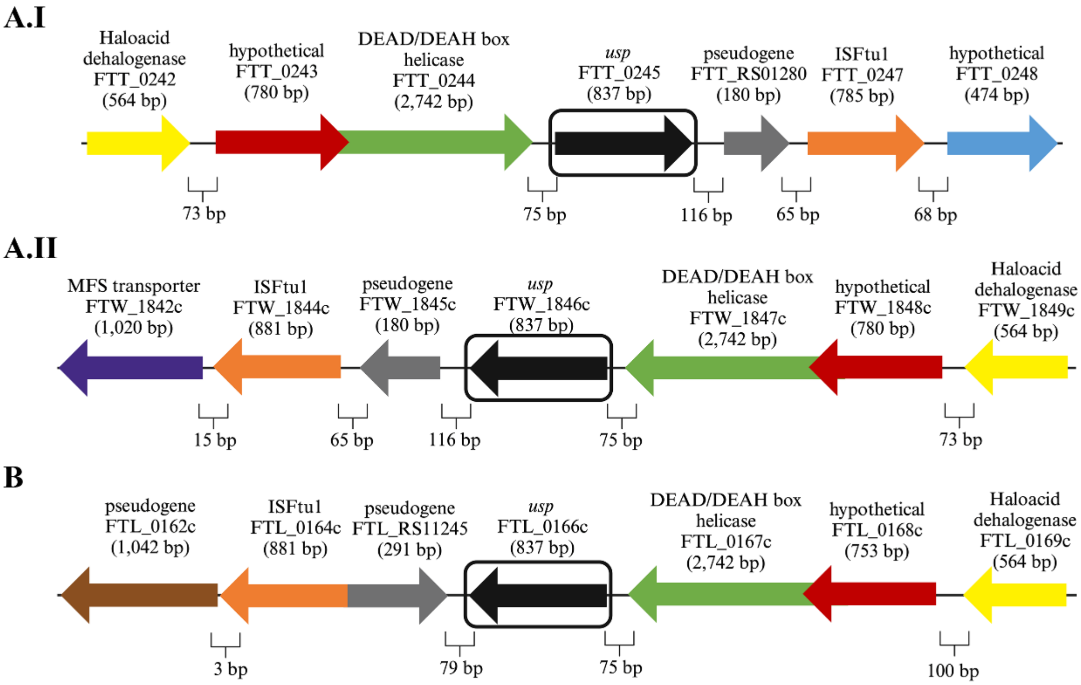

FIG S2 Protein alignment of universal stress protein (Usp) from representative strains from each *F. tularensis* subpopulation. These *F. tularensis* strains included subtype A.I strains SCHU S4 and MA00-2987, subtype A.II strains WY96-3418 and WY-00W4114, and type B strains FSC200 and LVS. Clustal Omega (version 1.2.4) was used to produce the protein alignment shown. Amino acid polymorphisms and cysteines are highlighted in yellow and grey, respectively.

```
B_LVS_Usp_FTL_0166                    MAYKKVLLAVNVYENADIVINSAVDFAKKNNTQVLKVVTVIDCVAPFAPSIVDFQHSIEQ 60
B_FSC200_Usp_FTS_0161                 MAYKKVLLAVNVYENADIVINSAVDFAKKNNTQVLKVVTVIDCVAPFAPSIVDFQHSIEQ 60
A.I_SCHU_S4_Usp_FTT_0245              MAYKKVLLAVNVYENADIVINSAVDFAKKNNTQVLKVVTVIDCVAPFAPSIVDFQHSIEQ 60
A.I_MA00-2987_Usp_FTZ_0228            MAYKKVLLAVNVYENADIVINSAVDFAKKNNTQVLKVVTVIDCVAPFAPSIVDFQHSIEQ 60
A.II_WY96-3418_Usp_FTW_1846           MAYKKVLLAVNVYENADIVINSAVDFAKKNNTQVLKVVTVIDCVAPFAPSIVDFQHSIEQ 60
A.II_WY-00W4114_Usp_FT4114_08990      MAYKKVLLAVNVYENADIVINSAVDFAKKNNTQVLKVVTVIDCVAPFAPSIVDFQHSIEQ 60
                                      ************************************************************

B_LVS_Usp_FTL_0166                    EAKEALDKLVTKISGIKVEHEVLVGNPAAEIVEYAEESNCDVIVLGSHATHGINLLLGSV 120
B_FSC200_Usp_FTS_0161                 EAKEALDKLVTKISGIKVEHEVLVGNPAAEIVEYAEESNCDVIVLGSHATHGINLLLGSV 120
A.I_SCHU_S4_Usp_FTT_0245              EAKEALDKLVTKISGIKVEHEVLVGNPAAEIVEYAEESNCDVIVLGSHATHGINLLLGSV 120
A.I_MA00-2987_Usp_FTZ_0228            EAKEALDKLVTKISGIKVEHEVLVGNPAAEIVEYAEESNCDVIVLGSHATHGINLLLGSV 120
A.II_WY96-3418_Usp_FTW_1846           EAKEALDKLVTKISGIKVEHEVLVGNPAAEIVEYAEESNCDVIVLGSHATHGINLLLGSV 120
A.II_WY-00W4114_Usp_FT4114_08990      EAKEALDKLVTKISGIKVEHEVLVGNPAAEIVEYAEESNCDVIVLGSHATHGINLLLGSV 120
                                      ************************************************************

B_LVS_Usp_FTL_0166                    ANAVLHKAKCDVLTVRVNDDENAYSKAHSYKRLLVPTDLENDSCVVVDKAKDIAKLYSAK 180
B_FSC200_Usp_FTS_0161                 ANAVLHKAKCDVLTVRVNDDENAYSKAHSYKRLLVPTDLENDSCVVVDKAKDIAKLYSAK 180
A.I_SCHU_S4_Usp_FTT_0245              ANTVLHKAKCDVLTVRVNDDENADSKAHSYKRLLVPTDLENDSYVVVDKAKDIAKLYSAK 180
A.I_MA00-2987_Usp_FTZ_0228            ANTVLHKAKCDVLTVRVNDDENADSKAHSYKRLLVPTDLENDSYVVVDKAKDIAKLYSAK 180
A.II_WY96-3418_Usp_FTW_1846           ANTVLHKAKCDVLTVRVNDDENADSKAHSYKRLLVPTDLENDSCVVVDKAKDIAKLYSAK 180
A.II_WY-00W4114_Usp_FT4114_08990      ANTVLHKAKCDVLTVRVNDDENADSKAHSYKRLLVPTDLENDSCVVVDKAKDIAKLYSAK 180
                                      **:******************** ******************* ****************

B_LVS_Usp_FTL_0166                    IDTAFVIPNDSISLMTYETDKFETKLDKFAEKNGITGEKSVMIGGISNSLLEKAAENKND 240
B_FSC200_Usp_FTS_0161                 IDTAFVIPNDSISLMTYETDKFETKLDKFAEKNGITGEKSVMIGGISNSLLEKAAENKND 240
A.I_SCHU_S4_Usp_FTT_0245              IDTAFVIPNDSISLMTYETDKVETTLDKFAEKNGITGEKSVMIGGISNSLLEKAAENKND 240
A.I_MA00-2987_Usp_FTZ_0228            IDTAFVIPNDSISLMTYETDKVETTLDKFAEKNGITGEKSVMIGGISNSLLEKAAENKND 240
A.II_WY96-3418_Usp_FTW_1846           IDTAFVIPNDSISLMTYETDKVETTLDKFAEKNGITGEKSVMIGGISNSLLEKAAENKND 240
A.II_WY-00W4114_Usp_FT4114_08990      IDTAFVIPNDSISLMTYETDKVETTLDKFAEKNGITGEKSVMIGGISNSLLEKAAENKND 240
                                      *********************.**.***********************************

B_LVS_Usp_FTL_0166                    LIVVGSHRRGAIGRFFLGSTANSILHQANVDVLVVRLK  278
B_FSC200_Usp_FTS_0161                 LIVVGSHRRGAIGRFFLGSTANSILHQANVDVLVVRLK  278
A.I_SCHU_S4_Usp_FTT_0245              LIVVGSHRRGAIGRFFLGSTANSILHQANVDVLVVRLK  278
A.I_MA00-2987_Usp_FTZ_0228            LIVVGSHRRGAIGRFFLGSTANSILHQANVDVLVVRLK  278
A.II_WY96-3418_Usp_FTW_1846           LIVVGSHRRGAIGRFFLGSTANSILHQANVDVLVVRLK  278
A.II_WY-00W4114_Usp_FT4114_08990      LIVVGSHRRGAIGRFFLGSTANSILHQANVDVLVVRLK  278
                                      **************************************
```

TABLE S1 Universal stress protein (Usp) homologs in microbes with or without conserved Walker A-like ATP-binding motifs

Bacterial species and Usp homolog (locus tag)[a]	Usp-like domain	Walker A-like motif GXXGX$_9$GS/T/N	ATP and/or cAMP binding	Reference/ Source
C. burnetii UspA (CBU_1916)	single	**G**SH**G**RHGIQLLL**G**S[b]	---	This study
C. burnetii UspA (CBU_1983)	single	**G**TH**G**HHGLDKLL**G**S[b]	---	This study
M. jannaschii Usp (MJ0577)	single	**G**SH**G**KTNLKEILL**G**S	yes (ATP)	(1)
M. tuberculosis Usp (Rv1636)	single	**G**NV**G**LSTIAGRLL**G**S	yes (cAMP > ATP)	(2)
M. tuberculosis Usp (Rv1996)	N-terminal<br>C-terminal	**G**YR**G**QGAVAGALL**G**S<br>**G**SH**G**RGGFPGMHL**G**S	---<br>---	(3)
M. tuberculosis Usp (Rv2005c)	N-terminal<br>C-terminal	**G**SS**G**RGALARGLL**G**S<br>**G**SH**G**RGGLTGMLL**G**S	---<br>---	(3)
M. tuberculosis Usp (Rv2026c)	N-terminal<br>C-terminal	**G**SQ**G**MGALGRLLL**G**S<br>**G**SH**G**RGGFSGMLL**G**S	---<br>---	(3)
M. tuberculosis Usp (Rv2028c)	N-terminal[c]	**G**AI**G**VHHFRPERV**G**S	---	(3)
M. tuberculosis Usp (Rv2319c)	N-terminal<br>C-terminal	**G**SFPSGRRARVLI**G**S[b]<br>**G**TSPFGDVARVFL**G**S[b]	---<br>---	This study
M. tuberculosis Usp (Rv2623)	N-terminal<br>C-terminal	**G**CL**G**SGRWPGRLL**G**S<br>**G**SR**G**RGGYAGMLV**G**S	yes (ATP)<br>yes (ATP)	(4)
M. tuberculosis Usp (Rv2624c)	N-terminal[c]	**G**SV**G**IGRYASSIL**G**S	---	(3)
M. tuberculosis Usp (Rv3134c)	N-terminal[c]	**G**SV**G**LDHVRGRR**G**S	---	(3)
S. enterica UspE (STM1661)	N-terminal<br>C-terminal	---[d]<br>**G**TV**G**RTGLSAAFL**G**N	no<br>yes (ATP)	(5)
H. influenzae UspA (HI_0815)	single	**G**HHQDFWSKLMS[e]	no	(6)
F. tularensis Usp (FTT_0245)	N-terminal<br>C-terminal	**G**SHATHGINLLL**G**S[b]<br>**G**SHRRGAIGRFFL**G**S[b]	---<br>---	This study

[a]Microbial Usp homologs were obtained from *Francisella tularensis* SCHU S4 (NCBI accession number NC_006570.2), *Coxiella burnetii* RSA 493 (NCBI accession number NC_002971.4), *Methanocaldococcus jannaschii* DSM 2661 (NCBI accession number NC_000909.1), *Mycobacterium tuberculosis* H37Rv (NCBI accession number NC_000962.3), *Salmonella enterica* LT2 (NCBI accession number NC_003197.2), and *Haemophilus influenzae* Rd KW20 (NCBI accession number L42023.1).
[b]Usp alignments were performed using ClustalW to identify regions with conserved or degenerated Walker A-like motifs.
[c]*M. tuberculosis* Usp with locus tags Rv2028c, Rv2624c,and Rv3134c contain two domains in which only the N-terminal domain is Usp-like.
[d]The N-terminal Usp-like domain in *S. enterica* UspE contains the residues MAHQHDRLEAVIFTP in the region where Walker A-like motifs are generally located in other Usp-like domains.
[e]Sequence shown for *H. influenzae* UspA is identical to the corresponding region in *Escherichia coli* UspA.

FIG S3 Assessment of the interaction between *F. tularensis* response regulator QseB and the *usp* promoter. Amplicons and recombinant protein samples were incubated for 30 minutes either at room temperature or on ice prior to the addition of loading buffer. The PCR-derived promoter region for *qseB* (lane 1) and *usp* (lane 2) without BSA, and PCR-derived promoter region for *qseB* (lane 3) and *usp* (lane 4) with 1 µg of BSA, after incubation at room temperature are shown. As positive controls, 1 µg of recombinant QseB and the associated *qseB* promoter region were incubated at room temperature (lane 5) and on ice (lane 6). The *usp* promoter region was mixed with 0.5 µg of recombinant QseB at room temperature (lane 7) and on ice (lane 10), 0.75 µg of recombinant QseB at room temperature (lane 8) and on ice (lane 11), and 1.0 µg of recombinant QseB at room temperature (lane 9) and on ice (lane 12). Concentrations of the amplicons containing the promoter region for *usp* and *qseB* remained constant at 500 ng. Results shown are from a representative experiment.

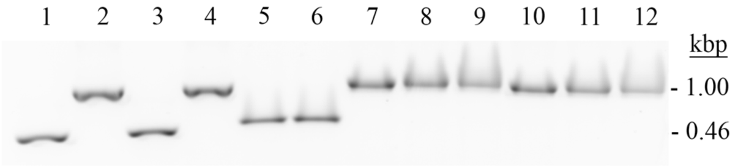

FIG S4 PCR amplification of *usp* chromosomal locus in *F. tularensis* wildtype and the isogenic Δ*usp* mutant. *F. tularensis* Δ*usp* mutants (lanes 1 and 2) and wildtype (lane 3) produced the expected 1,203 bp and 3,268 bp amplicons, respectively. No template control (lane 4) and DNA marker (lane 5) are also shown.

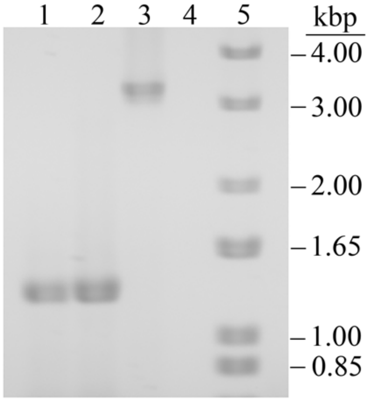

FIG S5 Catalase activity in *F. tularensis* wildtype and the isogenic Δ*usp* mutant in the absence and presence of paraquat treatment. **(A)** *F. tularensis* wildtype (WT) and Δ*usp* were either untreated or treated with 50 µM paraquat (PQ) for 1 hour and then evaluated for catalase activity by fractionation in a 12% precast native gel and subsequent staining with ferric chloride and potassium ferricyanide in the presence of hydrogen peroxide. Lane 1 contained 0.4 units of bovine catalase at 0.129 U/µL (positive control). Lanes 2 through 5 contained 0.5 µg of protein lysate from untreated WT (lane 2, control), paraquat treated WT (lane 3), untreated Δ*usp* (lane 4), and paraquat treated Δ*usp* (lane 5). **(B)** Graph depicts catalase activity in untreated and paraquat treated WT and Δ*usp* that was measured using a spectrophotometer-based assay. Data represents the mean ± SEM from three independent experiments and results were evaluated using unpaired *t* tests. No significant difference in catalase activity was obtained from these statistical analyses since *P* values were > 0.05.

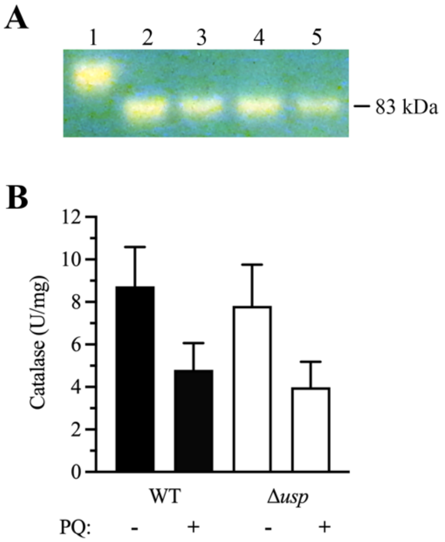

TABLE S2 Primers used for this study

Gene	*F. tularensis* SCHU S4 locus tag	Forward or primary primer (5' to 3')	Reverse or nested primer (5' to 3')	Amplicon size (bp)	Experimental use(s)
usp	FTT_0245	TAATGAGCTTGCATGCCTGCACTCATGGATTAGCGATGTATG	GCTACGTCCGAGTTATTATTATGCTAATAAAACCTTTTTGTACGCCAT	554	Δ*usp* primer set 1
usp	FTT_0245	TAATAATAACTCGGACGTAGCCTACTGCTTTAGCATTAGCAG	TTACGAATTCGAGCTCGGTACCCGAAGTACTTTGTCAATCTCGCAG	628	Δ*usp* primer set 2
usp	FTT_0245	GATAATAGTTATGATAAGGACGAGC	TGACTCGACACTAGTGCTAAG	2,440	Δ*usp* confirmation
usp	FTT_0245	CGTGGTACCAAAAATAAAATTTGATGTTTAATTAAATGCTTTTTTAT	TATGGATCCTTATTATTTAAGCCTTACAACCAAAACAT	933	Complementation plasmid with *usp* promoter
usp	FTT_0245	CTCCCAATAGCTCCTCTCCTATGGC	GCATATTCAACTATCTCTGCTGCCGG	503	5' RACE
usp	FTT_0245	TCTCCATGGCGTACAAAAAGGTTTTATTAGC	ATCTCTCGAGTTTAAGCCTTACAACCAAAACATC	837	rUsp/His_6
qseB	FTT_1557c	TCTCCATGGGTAGAATATTGTTGGCTGAAGATGATC	ATCTCTCGAGCTTAATTACTTTATCCTTTTGTACAAAG	690	rQseB/His_6
usp	FTT_0245	CTATAACTTTATTTCTCCAGAAGTTATCT	CATTAGGAATCTCCCTACTTACTTT	1000	EMSA
qseB	FTT_1557c	GACATGTTATAATTGACCTAGATTATTAG	CATACCAATATCTAAGACGACTATATC	458	EMSA
usp	FTT_0245	GTAGGGAGATTCCTAATGGCG	CTGTTTGCGGTTGAGCCCAAGAAG	803	mRNA stability
usp	FTT_0245	TGAAAGTTGTCACAGTTATTGACTG	CGATCACATCACAGTTACTTTCTTC	207	RT-qPCR
sspA	FTT_0458	GATAATGAGTGGTATCCAGTATTAG	AATCTGCTAAAGTAAAGCCAGAAG	163	RT-qPCR
oxyR	FTT_0556c	GCTGAGCTACTCTTAGAGAATG	CAGTAGGAGTTGCTAGTAAAGC	208	RT-qPCR
katG	FTT_0721c	GGAATGAAGCCTATAGGGTTTG	CTAGCTGCACCTTTGATATCAG	206	RT-qPCR
sodB	FTT_0068	CTTACGCTGTTGATGCATTAGAG	CTTGAGCAGCGTTATTAAATATTCC	186	RT-qPCR
furA	FTT_0030c	ACTCAACCTCGTGTTGAGATAC	CTCACCTTGATTTAACTCATACATTAC	211	RT-qPCR
lpnA	FTT_0901	CACAAGGAAGTGTAAGATTACAATG	TGAATCAGAAGCGATTACTTCTTTG	197	RT-qPCR normalization

Supplemental Data References

1. Zarembinski TI, Hung LW, Mueller-Dieckmann HJ, Kim KK, Yokota H, Kim R, Kim SH. 1998. Structure-based assignment of the biochemical function of a hypothetical protein: a test case of structural genomics. Proc Natl Acad Sci U S A 95:15189-15193.
2. Banerjee A, Adolph RS, Gopalakrishnapai J, Kleinboelting S, Emmerich C, Steegborn C, Visweswariah SS. 2015. A universal stress protein (USP) in mycobacteria binds cAMP. J Biol Chem 290:12731-12743.
3. O'Toole R, Williams HD. 2003. Universal stress proteins and *Mycobacterium tuberculosis*. Res Microbiol 154:387-392.
4. Drumm JE, Mi K, Bilder P, Sun M, Lim J, Bielefeldt-Ohmann H, Basaraba R, So M, Zhu G, Tufariello JM, Izzo AA, Orme IM, Almo SC, Leyh TS, Chan J. 2009. *Mycobacterium tuberculosis* universal stress protein Rv2623 regulates bacillary growth by ATP-binding: requirement for establishing chronic persistent infection. PLoS Pathog 5:e1000460.
5. Bangera M, Panigrahi R, Sagurthi SR, Savithri HS, Murthy MRN. 2015. Structural and functional analysis of two universal stress proteins YdaA and YnaF from *Salmonella typhimurium*: possible roles in microbial stress tolerance. J Struct Biol 189:238-250.
6. Sousa MC, McKay DB. 2001. Structure of the universal stress protein of *Haemophilus influenzae*. Structure 9:1135-1141.
